# Adipose triglyceride lipase promotes prostaglandin-dependent actin remodeling by regulating substrate release from lipid droplets

**DOI:** 10.1242/dev.201516

**Published:** 2023-06-08

**Authors:** Michelle S. Giedt, Jonathon M. Thomalla, Roger P. White, Matthew R. Johnson, Zon Weng Lai, Tina L. Tootle, Michael A. Welte

**Affiliations:** ^1^ Anatomy and Cell Biology, University of Iowa Carver College of Medicine, Iowa City, IA 52242, USA; ^2^ Department of Biology, University of Rochester, Rochester, NY 14627, USA; ^3^ Harvard T.H. Chan Advanced Multi-omics Platform, Harvard T.H. Chan School of Public Health, Boston, MA 02115, USA

**Keywords:** Actin cytoskeleton, Lipid droplets, Prostaglandin signaling, *Drosophila*, Oogenesis

## Abstract

Lipid droplets (LDs), crucial regulators of lipid metabolism, accumulate during oocyte development. However, their roles in fertility remain largely unknown. During *Drosophila* oogenesis, LD accumulation coincides with the actin remodeling necessary for follicle development. Loss of the LD-associated Adipose Triglyceride Lipase (ATGL) disrupts both actin bundle formation and cortical actin integrity, an unusual phenotype also seen when the prostaglandin (PG) synthase Pxt is missing. Dominant genetic interactions and PG treatment of follicles indicate that ATGL acts upstream of Pxt to regulate actin remodeling. Our data suggest that ATGL releases arachidonic acid (AA) from LDs to serve as the substrate for PG synthesis. Lipidomic analysis detects AA-containing triglycerides in ovaries, and these are increased when ATGL is lost. High levels of exogenous AA block follicle development; this is enhanced by impairing LD formation and suppressed by reducing ATGL. Together, these data support the model that AA stored in LD triglycerides is released by ATGL to drive the production of PGs, which promote the actin remodeling necessary for follicle development. We speculate that this pathway is conserved across organisms to regulate oocyte development and promote fertility.

## Teaser

**Summary:** Genetic and lipid studies reveal that the lipid droplet-associated Adipose Triglyceride Lipase provides the substrate for prostaglandin production during *Drosophila* follicle development, ultimately regulating actin remodeling and promoting fertility.

## INTRODUCTION

Rates of infertility are increasing (Sun et al., 2019). Fertility requires the production of high-quality oocytes. Among key factors ensuring oocyte quality are the amount and types of lipids present during oogenesis (Dunning et al., 2014; Brusentsev et al., 2019). In mammalian follicles, fatty acids (FAs) likely contribute a crucial source of energy as inhibitors of FA oxidation impair oocyte maturation (Dunning et al., 2010, 2011). Lipid signaling molecules – from steroid hormones to eicosanoids – control diverse aspects of oocyte development and fertility (Prates et al., 2014). Prostaglandins (PGs), a class of eicosanoids, regulate oocyte development, ovulation, fertilization, implantation, maintenance of pregnancy, and childbirth (Tootle, 2013; Sugimoto et al., 2015). Thus, control of lipid metabolism is central for oocyte development and fertility, yet few underlying mechanisms are known.

Key regulatory steps in lipid metabolism are mediated by lipid droplets (LDs), cellular organelles that store neutral lipids (Walther and Farese, 2012). LDs stockpile excess amounts of cholesterol and related sterols as sterol esters, and safely sequester toxic FAs in the form of triglycerides. Such FAs are released by LD-bound lipases to be shuttled to mitochondria for oxidative breakdown, used to generate membrane precursors, or turned into signaling molecules (Welte, 2015). Given that both triglycerides and LDs are abundant in oocytes, LDs may perform similar roles during oogenesis. Indeed, LD accumulation, composition and localization are dynamic during oocyte maturation across organisms (Ami et al., 2011; Dunning et al., 2014; Brusentsev et al., 2019), and, in the context of obesity, changes in oocyte LDs are associated with infertility (Jungheim et al., 2010; Cardozo et al., 2011; Marei et al., 2020). However, the functions of LDs in oogenesis remain largely undefined.

**Fig. 1.**
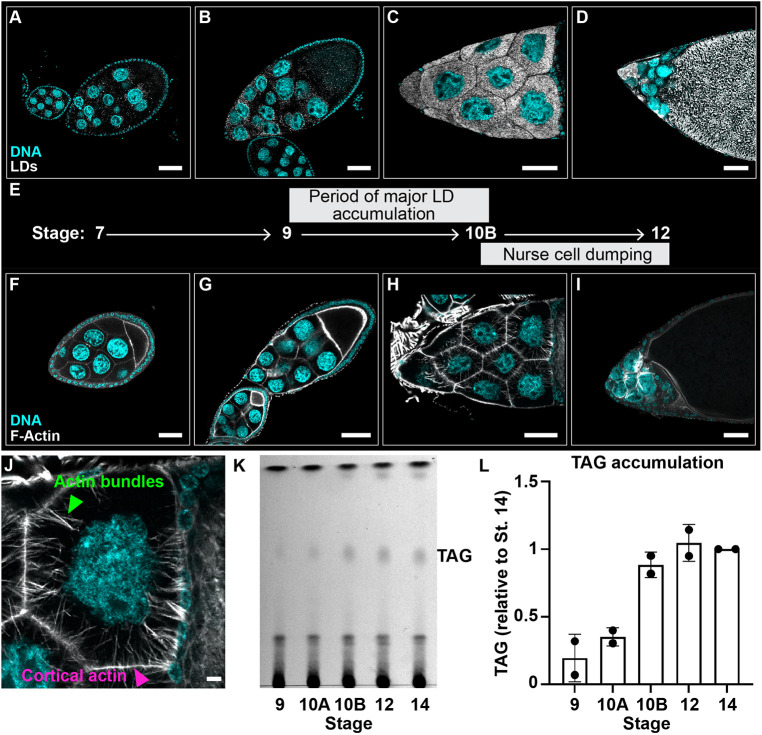
**LD accumulation and actin cytoskeletal remodeling occur during mid-oogenesis.** (A-I) Single confocal slices of wild-type (Oregon R) follicles of the indicated stages (E) stained for LDs (Nile Red, A-D) or F-Actin (phalloidin, F-I) in white, and DNA (Hoechst) in cyan. Scale bars: 50 µm. (E) Schematic depicting the progression of LD accumulation and actin remodeling during oogenesis. All of the images and the schematic in A-I are positioned on top of a black background. (J) Confocal slice of an S10B nurse cell stained for F-Actin (phalloidin) in white and DNA (Hoechst) in cyan; arrowheads indicate actin bundles (green) and cortical actin (magenta). Scale bar: 10 µm. (K) Thin layer chromatograph of whole-lipid extracts from late-stage follicles. (L) Quantification of TAG levels in K plotted relative to S14; error bars represent s.d. LDs begin to accumulate during S9 (B), are present in large numbers and evenly distributed throughout the nurse cell cytoplasm in S10B (C), are dumped into the oocyte during S11 and are highly abundant in the ooplasm by S12 (D). This temporal accumulation is reflected by increases in triglycerides and sterol esters (K,L), neutral lipids stored in LDs. Massive remodeling of the nurse cell actin cytoskeleton begins when LDs are highly abundant (E-H). The cortical actin thickens and actin bundles extend from the membrane to the nucleus during S10B (H). During S11, actin bundles hold the nurse cell nuclei in place while the cortical actin contracts, squeezing the cytoplasm into the oocyte (nurse cell dumping), causing oocyte expansion (I).

A promising model for uncovering the roles of LDs is *Drosophila* oogenesis. Adult female flies have two ovaries, each comprising ∼15 ovarioles or chains of sequentially maturing follicles, also called egg chambers. Each follicle is made up of ∼1000 epithelial follicle cells and 16 germline cells (15 nurse cells and one oocyte). Follicles develop over the course of ∼10 days through 14 stages. In mid-oogenesis, LDs undergo dramatic changes (Fig. 1A-D) (Buszczak et al., 2002). Prior to Stage 8 (S8), only a few scattered LDs are found throughout the follicle. In S9, LD biogenesis is massively upregulated in the nurse cells, so that by S10B the nurse cell cytoplasm is full of uniformly sized LDs (∼0.5 µm in diameter) (Buszczak et al., 2002; Teixeira et al., 2003). Thus, in just a few hours, nurse cells form tens of thousands of LDs. During S11, the LDs are transferred into the oocyte in a process termed nurse cell dumping, in which cytoplasmic contents of the nurse cells are squeezed into the oocyte through intercellular bridges called ring canals. These LDs provide the future embryo with stores of energy and specific proteins needed for development (Welte, 2015); indeed, embryos with reduced LDs have decreased hatching probability (Teixeira et al., 2003; Parra-Peralbo and Culi, 2011). Whether these LDs only provision the embryo or if they play roles in follicle development remains unclear.

One potential role of LDs during oogenesis may be to regulate lipid signaling. One class of lipid signaling molecules, PGs, plays crucial roles during mid-oogenesis. PGs are derived from arachidonic acid (AA), a poly-unsaturated FA, which is converted first into PGH_2_ and then immediately processed into one of several types of bioactive PGs (Tootle, 2013). These enzymatic steps are mediated by cyclooxygenase (COX) enzymes and specific PG synthases, respectively. *Drosophila* has a single COX-like enzyme called Pxt, and its absence results in severe defects in the actin remodeling necessary for late-stage follicle morphogenesis; this ultimately causes female sterility (Tootle and Spradling, 2008; Spracklen and Tootle, 2015). During S10B, the nurse cell actin cytoskeleton is remodeled: parallel actin bundles extend from the plasma membrane to the nuclei to form a cage, and the cortical actin substantially thickens (Fig. 1F-J). Both of these actin structures are required during S11 for nurse cell dumping; the cortical actin provides contractile force and the actin bundles prevent nuclei from being pushed into the ring canals and plugging them (Wheatley et al., 1995; Guild et al., 1997; Huelsmann et al., 2013). Loss of Pxt results in severe disruption in actin bundle formation, breakdown of cortical actin, and failure of nurse cell contraction (Tootle and Spradling, 2008; Spracklen and Tootle, 2015). Genetic studies have revealed that PG signaling regulates the activity of actin-binding proteins, including Fascin (Singed) and Enabled, to drive the actin remodeling necessary for follicle morphogenesis (Groen et al., 2012; Spracklen et al., 2014, 2019). However, how PG production is temporally and spatially regulated during *Drosophila* oogenesis remains unknown, but in many cells the release of AA from cellular lipids is the rate-limiting step (Funk, 2001; Tootle, 2013). Those precursor lipids are primarily thought to be phospholipids in cellular membranes, but in mammalian immune cells neutral lipids are a source of AA for PG synthesis (Dichlberger et al., 2014; Schlager et al., 2015). This raises the question of whether LD accumulation during *Drosophila* mid-oogenesis supports PG synthesis.

We sought to determine whether LDs contribute to *Drosophila* follicle development. Loss of the LD-associated Adipose Triglyceride Lipase (ATGL; Brummer) results in *pxt-*like actin remodeling defects during S10B. The similar phenotypes led us to ask whether ATGL and Pxt function in the same pathway during *Drosophila* oogenesis. Dominant genetic interaction and rescue experiments indicate that ATGL acts upstream of PG synthesis to drive actin remodeling. Further, lipidomic and exogenous AA experiments lead to the model that AA is stored in LDs, and ATGL is required to release AA from LDs to provide the substrate for PG production. Ultimately, these PGs control actin remodeling and thereby follicle development. These studies uncover new roles for LDs in regulating oogenesis, to sequester a developmentally important molecule, AA, and to control its release to drive specific processes.

## RESULTS

### ATGL is required for actin remodeling in nurse cells

During *Drosophila* oogenesis, LDs undergo dramatic changes in just a few hours (Fig. 1A-E). LDs start to accumulate at S9, fill the nurse cells by S10B, and are transferred to the oocyte by S12. In most tissues, the main neutral lipids stored in LDs are triglycerides and/or sterol esters (Walther and Farese, 2012). Using thin-layer chromatography, we found that developing follicles accumulate both classes of neutral lipids and triglyceride accumulation measured biochemically mirrors LD accumulation detected by microscopy (Fig. 1K,L).

**Fig. 2.**
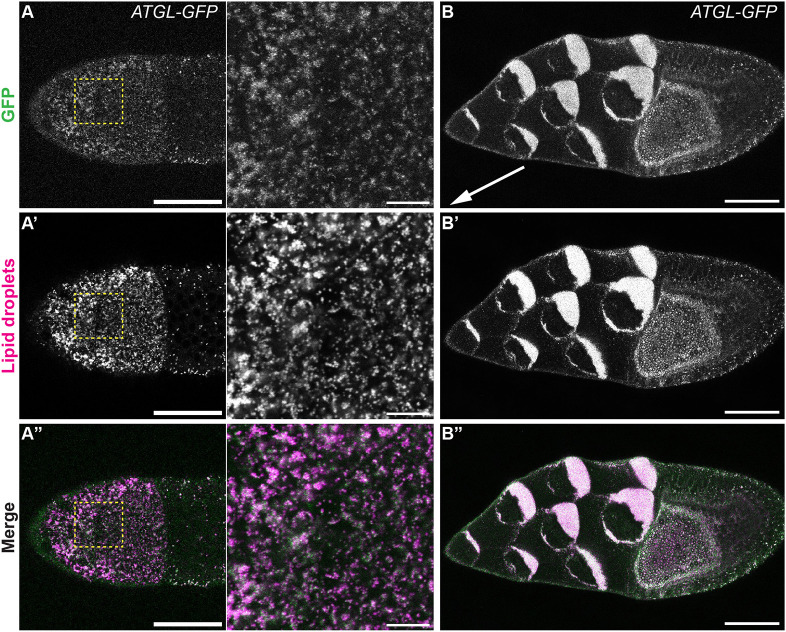
**ATGL is expressed in S10B nurse cells and localizes to lipid droplets.** (A-A″) Confocal slice of ATGL-GFP (green in merge) expressing S10B follicle stained for LDs (LipidSpot, magenta in merge); boxed regions are shown at higher magnification on the right. Note that a focal plane near the nurse cell surface was chosen as LD density there is lower than in the center, making colocalization easier to judge. (B-B″) Confocal slice of S10B centrifuged follicle expressing ATGL-GFP (green in merge) stained for LDs (LipidSpot, magenta in merge). Orientation during centrifugation is indicated by the arrow (which points towards the center of rotation). ATGL localizes to lipid droplets in S10B follicles (A-B″). Scale bars: 50 µm (main panels); 10 µm (right-hand panels in A-A″).

Previous *in situ* hybridization experiments found that *ATGL* is highly expressed in nurse cells during mid-oogenesis, just as LDs accumulate (Jambor et al., 2015). As ATGL catalyzes the conversion of triacylglycerol to diacylglycerol and free FA (Grönke et al., 2005), this pattern raises the possibility that LD triglycerides are broken down during these stages. Using an endogenously tagged *ATGL-GFP* allele (Zhao et al., 2022), we confirmed that ATGL is expressed in S10B follicles and colocalizes with LDs (Fig. 2A-A″). We also separated LDs from other cellular components by *in vivo* centrifugation: when living *Drosophila* follicles are centrifuged, their contents separate by density within each nurse cell/oocyte, with LDs accumulating on the side that faced up during centrifugation (Cermelli et al., 2006; Li et al., 2012). Staining of fixed centrifuged S10B follicles revealed that ATGL-GFP is enriched in the LD layer (Fig. 2B-B″).

**Fig. 3.**
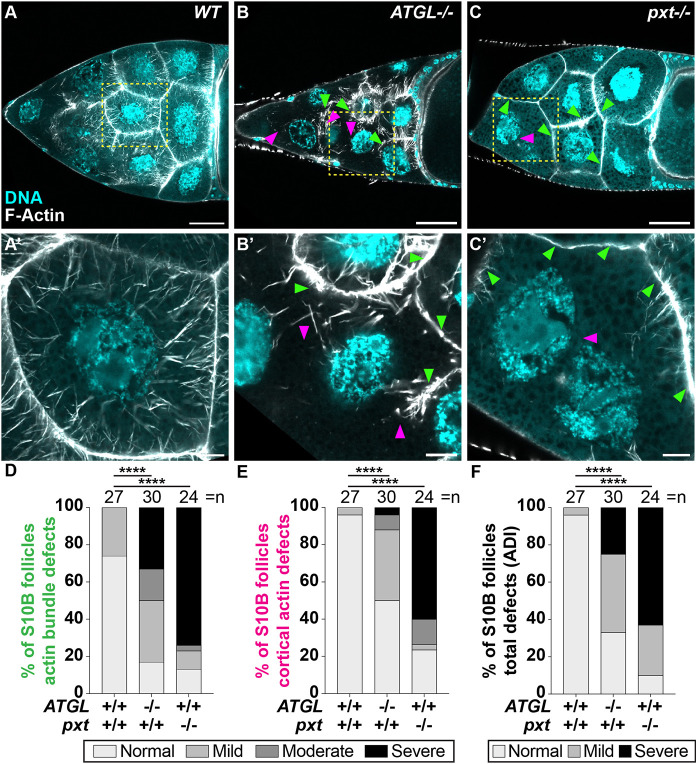
**ATGL regulates actin bundling and cortical actin integrity in S10B follicles.** (A-C′) Confocal slices of S10B follicles stained for F-Actin (phalloidin) in white, and DNA (DAPI) in cyan; yellow boxed regions are shown at higher magnification in A′-C′. Arrowheads indicate examples of actin bundling (green) and cortical actin (magenta) defects. Scale bars: 50 µm (A-C); 10 µm (A′-C′). (A,A′) WT, wild type (*yw*). (B,B′) *ATGL^−/−^* (*bmm^1^/bmm^1^*). (C,C′) *pxt^−/−^* (*pxt^f01000^*/*pxt^f01000^*). C′ was taken in a different focal plane to improve visualization of the defects. (D-F) Quantification of actin phenotypes from the following genotypes: WT, *yw*; *ATGL^−/−^*, *bmm^1^/bmm^1^*; *pxt^−/−^*, *pxt^f01000^/pxt^f01000^*; *pxt^EY03052^/pxt^EY03052^*. Actin bundles (D) and cortical actin (E) phenotypes were scored as normal, or mild, moderate or severe defects. Scores in D,E were summed and the total binned into three actin defect index (ADI) categories: normal, mild and severe. For a detailed description of actin scoring, please see Materials and Methods and Fig. S1. ****P<0.0001, Pearson's Chi-squared test. Error bars represent s.d. In wild-type late-S10B follicles, actin bundles extend from the nurse cell periphery to the nucleus, and the cortical actin is thickened (A,A′). Actin bundles fail to form or form improperly, and cortical actin is disrupted upon loss of ATGL (B,B′,D-F) or Pxt (C,C′,D-F).

As LD accumulation coincides with PG-dependent actin remodeling during S10B (Fig. 1E), we investigated whether ATGL has a role in these events. We found that females lacking ATGL display two types of actin defects (Fig. 3). First, actin bundles are aberrant. Some nurse cell membranes lack bundles, and the bundles that form are short, abnormally distributed, and/or fail to project toward the nuclei (Fig. 3B,B′, green arrowheads). Second, cortical actin is broken down, leading to the appearance of multinucleate nurse cells (Fig. 3B,B′, magenta arrowheads). To quantify these defects, we acquired confocal stacks of phalloidin-stained S10B follicles and separately scored actin bundles and cortical actin as having no (normal), mild, moderate or severe defects. We then summed these scores to give an actin defect index (ADI) to classify each follicle into three categories: normal, mild defects or severe defects (see Materials and Methods and Fig. S1 for details). Loss of ATGL resulted in actin bundle defects in ∼83% of the follicles (Fig. 3D) and cortical actin breakdown in ∼50% (Fig. 3E), with about a quarter of follicles exhibiting a severe ADI (Fig. 3F).

This combination of actin defects is rarely seen, as most actin regulators impact either actin bundle formation or cortical actin, but not both (Wheatley et al., 1995; Buszczak and Cooley, 2000). However, the same combination of phenotypes was observed when PG signaling is lost: lack of the COX-like enzyme Pxt caused collapsed, stunted or absent actin bundles, and cortical actin breakdown with a failure of nurse cell contraction (Fig. 3C,C′) (Tootle and Spradling, 2008; Spracklen and Tootle, 2015). Loss of Pxt resulted in actin bundle defects in ∼87% of the follicles and cortical actin breakdown in ∼76% (Fig. 3D,E), corresponding to a severe ADI for over 63% of the follicles (Fig. 3F). Thus, loss of Pxt and ATGL results in the same unusual combination of phenotypes.

### Pxt functions in a linear pathway with ATGL to regulate actin remodeling

**Fig. 4.**
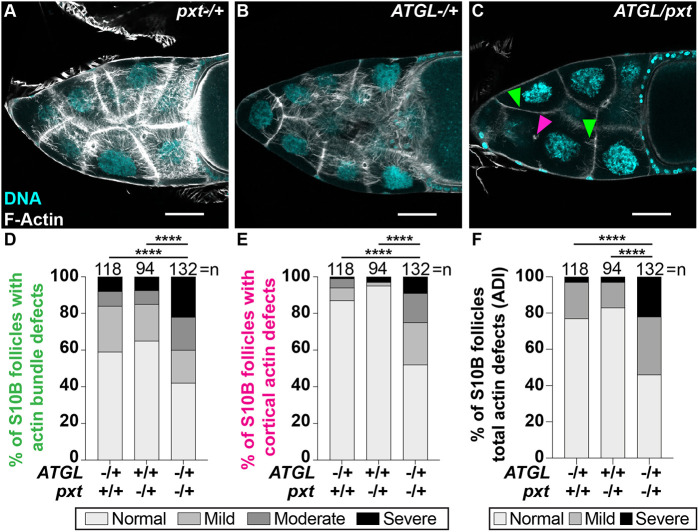
**ATGL acts in a linear pathway with Pxt to regulate actin remodeling.** (A-C) Maximum projections of three confocal slices of S10B follicles stained for F-Actin (phalloidin) in white, and DNA (DAPI) in cyan. Arrowheads indicate examples of actin bundling (green) and cortical actin (magenta) defects. Images were brightened by 30% to increase clarity. Scale bars: 50 μm. (A) *pxt*^−/+^ (*pxt^f01000^*/+). (B) *ATGL*^−/+^ (*bmm^1^*/+). (C) *ATGL/pxt* (*bmm^1^/pxt^f01000^)*. (D-F) Quantification of actin bundling (D), cortical actin (E) and ADI (E) as described for Fig. 3D-F from the following genotypes: *pxt*^−/+^, *pxt^f01000^*/+ and *pxt^EY03052^*/+; *ATGL*^−/+^, *bmm^1^*/+; *ATGL/pxt*, *bmm^1^*/*pxt^f01000^* and *bmm^1^*/*pxt^EY03052^*. ****P<0.0001, Pearson's Chi-squared test. Error bars represent s.d. Actin bundles and cortical actin integrity are largely normal in *pxt^−/+^* and *ATGL^−/+^* follicles (A,B,D-F). In contrast, in *ATGL*/*pxt* follicles actin bundles are absent, sparse and stunted, and there are instances of cortical actin breakdown (C-F).

The similar actin phenotypes in *pxt* (*Pxt*; FBgn0261987) and *ATGL* mutants suggest they may act in the same pathway to regulate actin remodeling. To test this hypothesis, we used a dominant genetic interaction assay, taking advantage of the fact that heterozygosity for the individual mutations (*pxt^−/+^* or *ATGL^−/+^*) has limited effects on actin remodeling (Fig. 4A,B). We can therefore use heterozygosity for *pxt* (*pxt^−/+^*) as a sensitized background to investigate whether reducing ATGL levels causes actin remodeling defects (Fig. S2). If ATGL and Pxt function in the same pathway, then double heterozygotes for *ATGL* and *pxt* (*ATGL/pxt*) will exhibit severe actin defects. Conversely, if they function in separate pathways, actin defects in the double heterozygotes will remain low or be additive with respect to the defects in the single heterozygotes. Strikingly, the double heterozygotes had severe actin defects (Fig. 4C-F). Single heterozygotes (*ATGL^−/+^* or *pxt^−/+^*) exhibited mild defects in actin bundles, with ∼7% being severe, whereas double heterozygotes (*pxt/ATGL*) exhibited ∼22% with severe bundle defects (Fig. 4D). Cortical actin defects were rare in the single heterozygotes, with over 87% being normal; conversely, only 52% of the double heterozygotes were normal (Fig. 4E). Using our ADI quantification, most heterozygous follicles were rated as normal (84% for *ATGL*^−/+^ and 87% for *pxt^−/+^*), whereas only 46% of the double heterozygotes exhibited normal actin remodeling; the frequency of severe cases increased from 3% in the single heterozygotes to 22% in the double heterozygotes (Fig. 4F). This synergistic increase in actin defects indicates that ATGL and Pxt regulate actin remodeling during S10B via a shared pathway.

### ATGL acts upstream of Pxt

Our genetic analysis indicates that ATGL and Pxt act in the same pathway to regulate actin remodeling but does not address whether ATGL acts upstream of Pxt or vice versa. For example, PGs produced by Pxt might control ATGL levels or promote binding of actin regulators to ATGL and thus to LDs; indeed, actin-binding proteins localize to LDs in various cell types (Pfisterer et al., 2017; Bersuker et al., 2018; Tan et al., 2021). Alternatively, ATGL might control Pxt expression or the availability of Pxt's substrate to ultimately control PG synthesis levels. Unfortunately, there is no available means of overexpressing ATGL in the germline to test whether it suppresses the actin defects in *pxt* mutants nor can we measure PG production in S10B follicles. PGs are produced at picogram levels and have extremely short half-lives (∼30 s); it is impossible to isolate enough tissue to analyze PGs by either enzyme-linked immunosorbent assay or high-performance liquid chromatography-mass spectrometry.

To circumvent these limitations, we took advantage of our *in vitro* egg maturation (IVEM) assay, in which isolated S10B follicles mature *in vitro* in a simple culture medium (Spracklen and Tootle, 2013). Maturation requires PG-dependent actin remodeling as both loss of Pxt or treatment with COX inhibitors, such as aspirin, impairs *in vitro* follicle development (Tootle and Spradling, 2008). In both cases, the block in development can be suppressed by exogenous PGF_2α_ (Tootle and Spradling, 2008); a stabilized PGF_2α_ analog, Fluprostenol (Flu), is used. This rescue is not complete, which could be due to PGF_2α_ not being provided at the correct level or time, or it could indicate that other PGs also contribute. In either case, if ATGL acts upstream of Pxt, PGF_2α_ should similarly suppress defects resulting from the loss of ATGL. If, in contrast, Pxt acts upstream of ATGL, PGF_2α_ should not modulate the *ATGL* mutant phenotype.

**Fig. 5.**
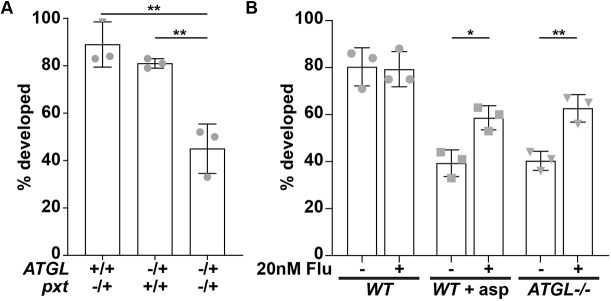
**ATGL functions upstream of PGF_2α_.** (A,B) Percentage of S10B follicles developing in the IVEM assay. In A, data are normalized to wild-type (*yw*) development for the following genotypes: *ATGL^−/+^* (*bmm^1^/+*), *pxt^−/+^* (*pxt^f01000^/+*) and *ATGL/pxt* (*bmm^1^/pxt^f01000^*). In B, wild-type (*yw*) and *ATGL^−/−^* (*bmm^1^/bmm^1^*) follicles were treated with control (ethanol), 1.5 mM aspirin in ethanol, and/or 20 nM PGF_2α_ analog (fluprostenol, Flu). *P<0.05, **P<0.005 (unpaired, two-tailed *t*-test). Error bars represent s.d. ATGL and Pxt interact genetically, as *ATGL/pxt* follicle development is reduced by ∼50% compared with the *ATGL^−/+^* and *pxt^−/+^* controls (A). Aspirin treatment or loss of ATGL reduces the percentage of follicles developing in control media. In both cases, this is suppressed by exogenously supplying a PGF_2α_ analog (B).

Using the IVEM assay, we first verified the genetic interaction between Pxt and ATGL. As expected, the majority of S10B follicles from single heterozygotes of *pxt* and *ATGL* developed *in vitro*, whereas over half of the follicles from double heterozygotes failed to develop (Fig. 5A). These data recapitulate what we observed when we quantified actin defects in the double heterozygotes (Fig. 4D-F). We then tested the role of PGF_2α_ downstream of ATGL. When treated with 1.5 mM aspirin, only ∼40% of wild-type S10B follicles developed, and this defect was partially suppressed by addition of PGF_2α_ (∼59% developing; Fig. 5B). Of the *ATGL* mutant follicles, only ∼40% developed, but addition of PGF_2α_ resulted in significant improvements (∼63% developing; Fig. 5B). Thus, the extent of the rescue was very similar for the two cases, suggesting that Pxt and PGF_2α_ act downstream of ATGL.

### AA is present in ovary triglycerides

How does ATGL function upstream of PGs? ATGL might regulate the expression of Pxt, but that is not the case, as loss of ATGL does not alter Pxt levels (Fig. S3). Studies in mammalian cells suggest an alternative possibility. There, ATGL (PNPLA2) releases AA from triglycerides stored in LDs to serve as the substrate for COX enzymes (Dichlberger et al., 2014). If ATGL similarly generates the substrate for Pxt, *Drosophila* follicles should have AA-containing triglycerides.

As flies are thought to lack the desaturases necessary to synthesize polyunsaturated fatty acids such as AA (Shen et al., 2010), AA in triglycerides is derived from the diet. We, therefore, fed flies food supplemented with fluorescently labeled AA for ∼12 h to assess AA localization in S10B follicles. AA became concentrated in puncta throughout the nurse cell cytoplasm; these structures are LDs, based on co-staining with a LD-specific dye and enrichment of AA in the LD layer of centrifuged follicles (Fig. S4). Thus, AA present in the diet can reach the ovary where it accumulates in LDs.

**Fig. 6.**
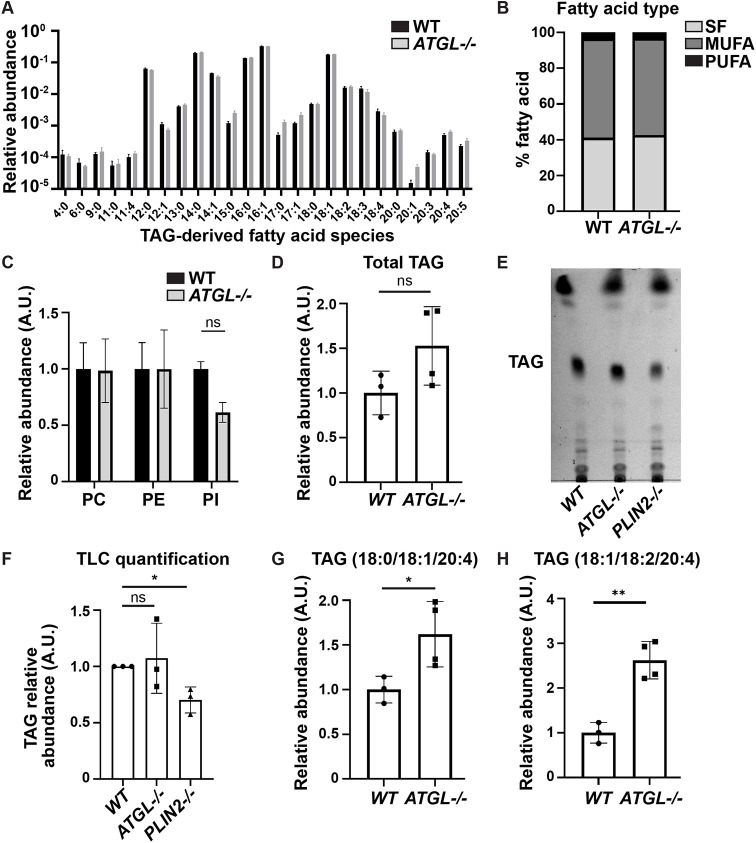
**Ovary triglycerides contain arachidonic acid.** (A-F) Lipids were extracted from wild-type (WT; Oregon R) and *ATGL^−/−^* (*bmm^1^/bmm^1^*) ovaries and analyzed by mass spectrometry. Error bars represent s.d. (A) Abundance of individual FAs in triglycerides (TAGs) expressed as fraction of all such FAs; Šídák's multiple comparisons test. (B) Total amount of saturated (SF), monounsaturated (MUFA) or polyunsaturated (PUFA) FAs relative to all FAs in TAGs; Tukey's multiple comparisons test. (C) Levels of the three phospholipid classes phosphatidylcholine (PC), phosphatidylethanolamine (PE) and phosphatidylinositol (PI); Šídák's multiple comparisons test. (D) Overall TAG levels. ns=*P*=0.1239 (two-tailed, unpaired *t*-test). (E) Thin layer chromatograph of whole-lipid extracts from S14 follicles of the indicated genotypes. (F) Quantification of TAG in E normalized to the wild type on each plate, *P=0.0111 (two-tailed, paired *t*-test). ns, not significant. (G,H) Quantification of two TAG species containing AA. *P=0.0419, **P=0.0019 (two-tailed, unpaired *t*-tests). A.U., arbitrary units. Wild-type and *ATGL* mutant ovaries exhibit a similar abundance of FAs in TAGs (A,B), phospholipids (C), and total TAGs (D) by lipidomic analysis (no significant differences detected). Similarly, thin layer chromatographic analysis of S14 follicles reveals no differences in TAGs (E,F). Two AA-containing TAG species are present in wild-type ovaries, and their levels are elevated in the absence of ATGL (G,H).

Next, we raised wild-type and *ATGL* mutant flies on regular food, extracted lipids from their ovaries, and analyzed those lipids by liquid chromatography with tandem mass spectrometry (LC-MS/MS). Among the 98 different triglyceride species identified (Table S1), two contained a 20-carbon FA with four double bonds (20:4), presumably AA. AA was relatively rare, making up in the order of 0.06-0.07% of all FAs detected in triglycerides (Fig. 6A), with four other FA species accounting for the bulk (∼85%) of FAs: the mono-unsaturated palmitoleic (16:1) and oleic (18:1) acids, and the saturated myristic (14:0) and palmitic (16:0) acids. We did not detect AA in the phospholipids identified in our analysis (Table S1).

The content of phospholipids (Fig. 6C) and total triglycerides (Fig. 6D) were not significantly different between wild-type and *ATGL* mutant ovaries. The overall FA profile in triglycerides was also similar between the two genotypes (Fig. 6A,B). In addition, there was no significant difference in total triglycerides in S14 oocytes, as detected by thin layer chromatography (Fig. 6E,F), even though we could confirm the previously described reduced triglyceride loading in our positive control, *PLIN2* (*Lsd-2*) mutants (Teixeira et al., 2003). Thus, ATGL-mediated lipolysis during oogenesis does not appear to lead to bulk turnover of LDs and may be restricted to supporting lipid signaling.

Supporting this possibility, wild-type and *ATGL* mutant ovaries exhibited differences in the AA-containing triglycerides, both of which were elevated in the absence of ATGL (Fig. 6G,H). This trend persisted even when measurements were normalized to all lipids in the sample (Fig. S5). These observations are consistent with the possibility that in the *ATGL* mutants less AA is released from triglycerides, resulting in a reduced pool of free AA available for signaling.

### ATGL promotes AA release from LDs

**Fig. 7.**
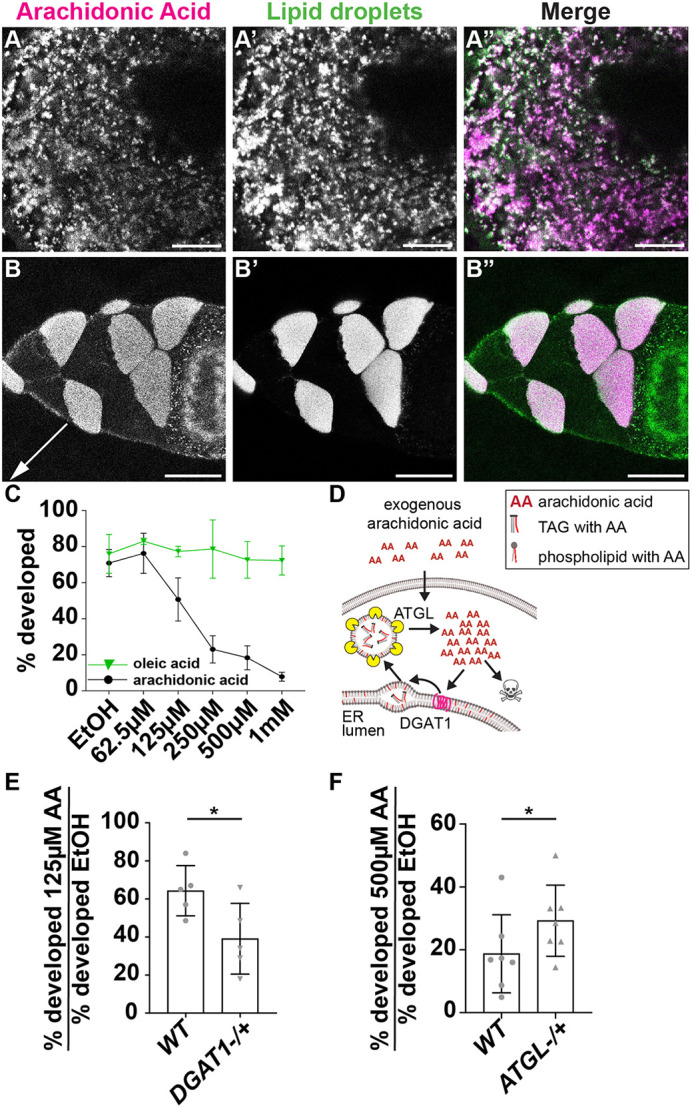
**ATGL regulates the release of arachidonic acid from lipid droplets.** (A-B″) Confocal slices of live (A-A″) or centrifuged (B-B″) wild-type (Oregon R, orientation during centrifugation indicated by arrow) follicles treated with NBD AA (green in merge) and LipidSpot (magenta in merge). Note that in A-A″ a focal plane near the nurse cell surface was chosen as LD density there is lower than in the center, making colocalization easier to judge. Scale bars: 10 µm (A-A″); 50 µm (B-B″). (C) Dose curve of the percentage of wild-type (*yw*) S10B follicles developing in the modified IVEM assay with increasing concentrations of AA (black circles and line) or oleic acid (green triangles and line). EtOH, ethanol. Error bars represent s.d. (D) Schematic depicting how LDs may buffer AA toxicity. Exogenous AA is rapidly sequestered into LDs by DGAT1, and ATGL can release AA from LD triglycerides. Too much free AA is toxic. (E,F) Percentage of S10B follicles developing in the modified IVEM assay for the indicated genotypes and conditions. Error bars represent s.d., *P<0.05 (two-tailed, paired *t*-test). In E, wild-type (WT, *yw*) and *DGAT1^−/+^* (*mdy^QX25^/+*) follicles were treated with 125 µm AA (intermediate dose). In F, wild-type (WT, *yw*) and *ATGL^−/+^* (*bmm^1^/+*) follicles were treated with 500 µm AA (high dose). AA is present in LDs, but AA uptake varies between LDs (A-B″). High levels of AA, but not oleic acid, suppress S10B follicle development (C). Reducing the level of DGAT1, which is expected to decrease AA incorporation into LDs, enhances the ability of an intermediate dose of AA to block *in vitro* development (E). Conversely, reducing ATGL suppresses the ability of a high dose of AA to block *in vitro* development (F).

Our lipidomic and imaging data suggest that AA is stored as triglycerides in LDs and its release from LDs is diminished or blocked in *ATGL* mutants. In an attempt to circumvent this block and directly provide free substrate for Pxt, we incubated isolated follicles with media containing fluorescently labeled AA. Monitoring AA localization by live imaging and *in vivo* centrifugation, we found that such exogenous AA predominantly accumulates in nurse cell LDs (Fig. 7A-B″). A similar pattern has also been observed in other systems, where exogenously applied AA is first routed to LDs (Weller and Dvorak, 1994; Bozza et al., 2009, 2011). One reason this may occur is that free AA is toxic at near physiological levels in many cells (Pompeia et al., 2003), and, thus, sequestering AA into LDs prevents free AA from reaching toxic levels.

If exogenous AA is similarly toxic to follicles, it will preclude its use to rescue PG production. We therefore assessed the dose-dependent effects of AA on wild-type S10B follicle development with a modified IVEM assay. One of the IVEM medium's key components is fetal bovine serum (FBS), which supplies growth factors and numerous nutrients, including unknown amounts of FAs such as AA. Thus, we reduced the amount of FBS from 10% to 2.5%, which allows most follicles to mature, and then assessed the effects of AA. At ∼60 µM AA, a slightly higher percentage of follicles developed than in control medium, but that fraction dropped steadily as AA concentrations were raised (Fig. 7C). Notably, oleic acid (OA) did not impair follicle development at any concentration (Fig. 7C). Thus, it is specifically free AA that is dangerous to the follicles, which provides a rationale for why free AA is rapidly sequestered into LDs.

Because exogenous AA is toxic and rapidly sequestered into LDs, using it as Pxt substrate is challenging as we cannot target it specifically to the site of PG synthesis. However, we reasoned that this toxicity could nevertheless be used as a tool to probe the functions of LDs and ATGL in regulating free AA levels.

Specifically, we investigated whether LD sequestration modulates AA toxicity. To be stored in LDs, free FAs must be incorporated into triglycerides. The final step of triglyceride synthesis is mediated by diacylglycerol O-acyl transferases (DGATs), which catalyze the attachment of a third FA to diacylglycerol. Like most animals, *Drosophila* encodes two such enzymes, DGAT1 (Midway) and DGAT2 (Yen et al., 2008). Genome-wide expression data indicate that DGAT1 predominates in most tissues, including in ovaries (Chintapalli et al., 2007). In *DGAT1* loss-of-function mutants, LDs fail to accumulate; these follicles die by S8/9 of oogenesis (Buszczak et al., 2002), although the underlying mechanism remains to be elucidated. If exogenous AA, as we hypothesize, is incorporated into LDs, then reducing DGAT1 levels should enhance AA toxicity (Fig. 7D). We tested this idea with our modified IVEM assay and treated follicles with 125 µM AA. At this concentration, 64% of wild-type follicles developed, whereas only 39% of *DGAT1^−/+^* follicles developed (Fig. 7E). As exogenous AA is predominately incorporated into LDs (Fig. 7A-B″), these observations are consistent with the model that exogenous AA toxicity is reduced by sequestration into LDs.

If ATGL releases AA from internal LD stores and thus generates free AA, decreasing ATGL levels should reduce the toxicity of high levels of exogenous AA, as total free AA levels will be lower (Fig. 7D). Indeed, twofold more S10B follicles from *ATGL* heterozygotes developed in the presence of 500 µM AA than wild-type follicles (Fig. 7F). Together, these findings support the model that AA is stored in LDs and ATGL is required to release AA from LDs. This AA can then be used for PG production, and thus to promote the actin remodeling necessary for follicle development.

### Pxt is not enriched on LDs

**Fig. 8.**
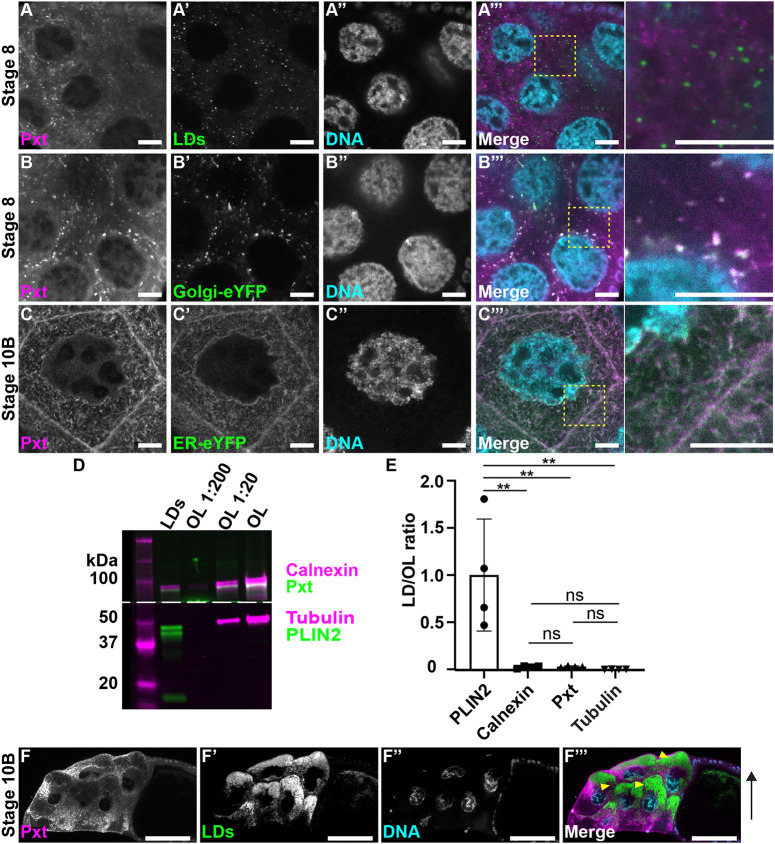
**Pxt is not enriched on lipid droplets.** (A-C‴) Confocal slice of wild-type (Oregon R) nurse cells of the indicated stages, stained for Pxt (A-C), organelle markers (A′-C′) and DNA (A″-C″; Hoechst). Merged images (A‴-C‴; boxed regions are shown at higher magnification on the right): Pxt, magenta; organelle marker, green; DNA, cyan. Organelle marker: LDs (A-A‴; Nile Red); Golgi-eYFP (B′-B‴); and ER-eYFP (C′-C‴). Scale bars: 10 µm. (D) Western blots of LDs purified from whole ovaries and a dilution series of ovary lysate (OL) for Calnexin (magenta; ER marker) and Pxt (green) in the top half of the blot and α-Tubulin (magenta, loading control) and PLIN2 (green; LD marker) in the bottom half of the blot; dashed line indicates where the membrane was cut. (E) Quantification of protein abundance in the LD fraction relative to undiluted OL, normalized to the average value for PLIN2. Error bars represent s.d. **P=0.0028, Tukey's multiple comparisons test. ns, not significant. (F-F‴) Confocal slice of centrifuged wild-type Stage 10B follicle stained for Pxt (F, magenta), LDs (F′, Nile Red, green), and DNA (F″, Hoechst, cyan). Orientation during centrifugation indicated by arrow. Arrowheads indicate the LD layer. Scale bars: 50 µm. Pxt does not colocalize with LDs (A-A‴), is not enriched on purified LDs (D,E), and does not co-accumulate with LDs in centrifuged follicles (F-F‴). Instead, Pxt localizes to the Golgi during S8 (B-B‴) and the ER during S10B (C-C‴).

How does ATGL provide AA to Pxt for PG synthesis? PG production occurs at the cellular location of the COX enzyme, as the active site of the enzyme faces the lumen of the organelle it localizes to. In other systems, components of the PG synthesis machinery are sometimes enriched on LDs, and, therefore, PG production could occur on LDs (Bozza et al., 2011). To determine whether this is true during *Drosophila* oogenesis, we used immunostaining, LD purification, and *in vivo* centrifugation to assess Pxt's relationship to LDs in wild-type nurse cells. All three methods showed that Pxt is not enriched on LDs (Fig. 8). Immunostaining revealed that Pxt localizes to the Golgi compartments prior to S9 and to the endoplasmic reticulum (ER) by S10B, but not obviously to LDs (Fig. 8A-C‴). Furthermore, Pxt localization was not affected by loss of ATGL (Fig. S6). We also prepared an LD-enriched biochemical fraction from ovary lysates, as previously described for embryos (Li et al., 2012), and analyzed it by western blotting for Pxt, PLIN2 (as a marker for LDs) and Calnexin (as marker for the ER). Pxt behaved like an ER protein, not like a LD protein (Fig. 8D,E). Finally, staining of fixed centrifuged follicles revealed no enrichment of Pxt in the LD layer (Fig. 8F-F‴). We conclude that the majority of Pxt is not on LDs. Thus, strong enrichment of the PG synthesis machinery on LDs is not required for LDs to play a role in PG production.

**Fig. 9.**
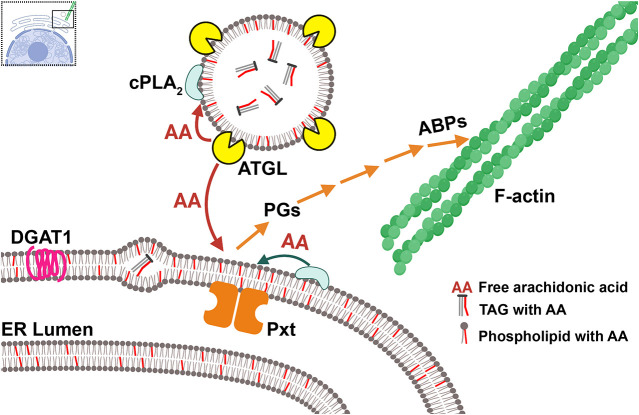
**ATGL regulates actin remodeling via prostaglandin signaling.** Schematic (created with BioRender.com) summarizing our findings. TAG (triglyceride) synthesized by DGAT1 (magenta) accumulates between the leaflets of the ER membrane and LDs bud from the ER (not shown). AA (red lines) is stored in TAG or phospholipids. Our data lead to the model that ATGL (yellow) liberates AA from LD TAGs, providing AA, either directly or indirectly, for Pxt to produce PGs to regulate the activity of actin-binding proteins (ABPs), including Fascin and Enabled (not shown), to drive actin remodeling. AA (red) freed from TAG by ATGL may be directly supplied to Pxt or first incorporated into phospholipids on LDs, ER or other membranes. Subsequently, cPLA2 (light blue) releases AA from those phospholipids, providing AA for PG production.

Together, our data lead to the model that during S10B, AA stored in LD triglycerides is released by ATGL and serves as the substrate for PGF_2α_ production by ER-localized Pxt. PGF_2α_ signaling then drives the actin remodeling necessary for late-stage follicle development (Fig. 9).

## DISCUSSION

Our data reveal that the LDs produced during *Drosophila* oogenesis are not just lipid and protein stores for the future embryo, but play a crucial regulatory role during follicle development. Loss of the triglyceride lipase ATGL results in cortical actin breakdown and defective actin bundle formation during S10B of oogenesis, an unusual combination of phenotypes also observed in flies lacking Pxt, the *Drosophila* COX-like enzyme (Tootle and Spradling, 2008). Dominant genetic interactions and PG treatment of follicles *in vitro* reveal that ATGL and Pxt act in the same pathway to regulate actin remodeling, with ATGL upstream of Pxt. Our lipidomic, fluorescent AA, and AA toxicity studies support the model that AA is stored in triglycerides in LDs, ATGL is required to release that AA, which is then used as substrate for Pxt-dependent PG production, and those PGs regulate the actin dynamics necessary for follicle development (Fig. 9). Although PG signaling is known to regulate actin remodeling across organisms and cell types (Peppelenbosch et al., 1993; Pierce et al., 1999; Dormond et al., 2002; Tamma et al., 2003; Bulin et al., 2005; Birukova et al., 2007), this study provides the first evidence linking LDs to PG-dependent actin remodeling.

### LDs provide the substrate for PG production

The enzymatic release of AA from cellular lipids is the rate-limiting step for PG production (Funk, 2001; Tootle, 2013). This free AA might originate from two different sources: phospholipids and triglycerides. In mammalian systems, both sources contribute to PG synthesis (Weller and Dvorak, 1994). Cytoplasmic phospholipase A2 (cPLA2) releases AA from phospholipids, and, in many cell types, inhibiting cPLA2 severely impairs PG production (Barbour and Dennis, 1993; Miyaura et al., 2003; Ghosh et al., 2004). Knockdown/inhibition of ATGL decreases PG production in mast cells (Dichlberger et al., 2014) and leukocytes (Schlager et al., 2015).

Our data support the model that in *Drosophila* follicles LD triglycerides are a source of AA for PG production and that ATGL is responsible for its release. AA from LDs might either directly serve as substrate for Pxt, or it might first be incorporated into phospholipids, to be subsequently released by cPLA2 (Fig. 9). Such models explain the genetic interaction between Pxt and ATGL (Figs 4 and 5A), the fact that exogenous PGF_2α_ can promote maturation of *ATGL* mutant follicles (Fig. 5B), the increase in AA-containing ovary triglycerides in *ATGL* mutants (Fig. 6G,H), and that reducing ATGL suppresses the lipotoxicity of exogenous AA (Fig. 7F). As the loss of Pxt causes more severe defects than the loss of ATGL, additional mechanisms of PG production likely exist. Perhaps ATGL and LDs initiates PG production that is later driven by cPLA2 or vice versa.

### Site of PG synthesis

Although LDs are a source of AA during *Drosophila* oogenesis, our data do not identify the site of PG production. In some mammalian cell types, the PG synthesis machinery localizes to LDs and produces PGs there (Yu et al., 1998; Accioly et al., 2008; Moreira et al., 2009; Bozza et al., 2011; D'Avila et al., 2011). However, immunofluorescence and biochemical purification show that Pxt is not enriched on LDs, but is present throughout the ER during S10B (Fig. 8). How, then, do LDs facilitate PG production?

LDs originate from the ER and, in many cells, retain physical connections with the ER after biogenesis (Walther and Farese, 2012; Olzmann and Carvalho, 2019); in particular, the phospholipid monolayer around LDs can remain continuous with one leaflet of the ER membrane (Cottier and Schneiter, 2022). Additional lipidic bridges between the ER and LDs can form transiently. These connections can mediate exchange of proteins and lipids (Salo et al., 2019; Cottier and Schneiter, 2022; Olarte et al., 2022) and thus presumably transfer AA freed from LDs. What remains unclear is how far free AA can diffuse (either via the cytosol or within membranes) before being metabolized; it might only be able to reach Pxt molecules present in ER regions immediately adjacent to LDs. Nevertheless, LD-derived AA could still reach distant regions of the ER by being incorporated into ER phospholipids, which can diffuse throughout the entire ER; in a second step, AA would then be released by cPLA2 to provide substrate for Pxt.

We find that LDs can function as important regulators of PG signaling even when the relevant COX enzyme are not enriched on LDs. This finding should prompt a careful re-examination of the functional impact of LDs in the many PG-producing cell types in which COX enzymes are distributed throughout the ER, nuclear envelope, and Golgi. Thus, LDs may regulate PG synthesis much more widely than previously recognized.

### Why LDs?

If AA released from phospholipids can serve as substrate for Pxt, why is AA incorporated into LDs at all? Although LDs may store AA for later use in embryogenesis, our experiments raise the additional possibility that LDs are needed to buffer the toxicity of free AA. We found that high levels of exogenous AA inhibit follicle development (Fig. 7C), similar to previous observations in bovine granulosa cells where AA induces apoptosis (Zhang et al., 2019). In fact, AA is cytotoxic for many cells, e.g. cultured hippocampal neurons (Okuda et al., 1994), even at concentrations that overlap physiological ones (Pompeia et al., 2003). This toxicity is likely due to the diverse roles of AA in cellular signaling, even beyond PG synthesis (Brash, 2001; Tallima and El Ridi, 2018). Because of such toxicity, levels of free AA are tightly regulated across organisms, often by turning excess AA into inert triglycerides stored in LDs. Indeed, in mammalian cells, exogenous AA is initially almost exclusively incorporated into LDs (Weller and Dvorak, 1994; Bozza et al., 2009, 2011). LDs likely have a similar buffering role in *Drosophila* follicles, as exogenous fluorescent AA rapidly localizes to LDs (Fig. 7A-B″) and triglyceride synthesis and breakdown modulate toxicity to exogenous AA (Fig. 7D-F).

Such an AA buffering function of LDs aligns with the recent recognition of LDs as a crucial way station during FA trafficking (Welte, 2015); they transiently sequester excess FAs from external and internal sources to prevent cellular damage, such as ER stress and mitochondrial dysfunction (Chitraju et al., 2017; Nguyen et al., 2017). Once safely stored in LDs, FAs can then be released in a regulated manner and directed to specific intracellular fates, such as energy production or signaling (Haemmerle et al., 2011; Rambold et al., 2015). Thus, LDs serve as FA trafficking hubs to both channel FAs to the correct intracellular destinations and buffer the FA supply.

### ATGL functions in development

Like in mammals, ATGL in flies is best known for its role in fat storage and energy homeostasis in adipose tissue (Grönke et al., 2005). ATGL is highly expressed in the larval and adult fat body; its absence causes triglyceride overaccumulation in this tissue, and overexpression depletes organismal fat stores (Grönke et al., 2005). Here, we show that ATGL has a key role in follicle development, via PG signaling and actin remodeling. ATGL has previously been implicated in other important developmental transitions, although the underlying mechanisms remain unclear. In male flies, ATGL in neurons and testes regulates whole-body energy storage by an as-yet-uncharacterized mechanism thought to involve systemic signaling (Wat et al., 2020). In mammalian muscle, ATGL in satellite cells is important for proper differentiation and efficient recovery from muscle injury (Yue et al., 2022). Finally, ectopic ATGL expression has beneficial roles in two *Drosophila* disease models: neurodegeneration due to mitochondrial dysfunction (Liu et al., 2015) and impairment of nephrocytes, components of the renal system, due to high-fat diet (Lubojemska et al., 2021). Our work identifies a specific signaling pathway, PG signaling, regulated by ATGL during oogenesis. It will be important to determine whether ATGL-based PG signaling facilitates the other developmental functions of ATGL.

### LDs as regulators of female fertility

Animal development requires careful regulation of metabolism, specific to tissue type and developmental stage (Sieber and Spradling, 2017). Yet, despite the fact that LDs have a central role in lipid metabolism and energy homeostasis in normal physiology and diseases (Walther and Farese, 2012; Hashemi and Goodman, 2015; Welte, 2015), they have only been implicated as regulators of development in a handful of cases (Chan et al., 2007; Li et al., 2012; Johnson et al., 2018; Stephenson et al., 2021; Yue et al., 2022). Our analyses not only reveal that LDs play a crucial role during *Drosophila* follicle development, but also identify PG signaling as one mechanism whereby LDs act.

We speculate that this pathway is conserved across organisms. Indeed, LD accumulation, composition and localization are dynamic during oocyte maturation (Ami et al., 2011; Dunning et al., 2014), LDs are hubs for FA trafficking, and FA levels (including that of AA) are important for oocyte development in many species (Dunning et al., 2014; Prates et al., 2014; Brusentsev et al., 2019). Furthermore, in mouse models and in humans, metabolic syndrome causes failures in oocyte maturation and decreases fertility (Wattanakumtornkul et al., 2003; Jungheim et al., 2010; Cardozo et al., 2011; Marei et al., 2020). Obesity directly impairs oocyte quality, as fertilized oocytes from non-obese donors can be successfully transplanted into obese recipients without affecting implantation rates or pregnancy success (Styne-Gross et al., 2005). In addition, it is well-established that PG signaling plays key roles in oocyte development (Akil et al., 1996; Smith et al., 1996; Lim et al., 1997; Pall et al., 2001; Wang et al., 2004; Takahashi et al., 2006, 2010). Recent evidence also implicates LDs as foci of PG production during reproduction. COX enzymes localize to LDs in the rat corpus luteum (Arend et al., 2004), and in fetal membranes during advanced gestation and at induction of labor, times when PG synthesis and signaling are upregulated (Meadows et al., 2003, 2005). Whether the effects of PG on oogenesis and reproduction in mammals are due to changes in the actin cytoskeleton is not yet known. However, cytoplasmic actin density and cortical actin thickness increase during oocyte maturation, contribute to meiotic resumption, and play roles in fertilization and early embryonic divisions (Coticchio et al., 2015; Namgoong and Kim, 2016). Given these data, and our findings connecting LDs, PG signaling and actin remodeling during *Drosophila* oogenesis (Fig. 9), we speculate that this pathway is conserved across organisms to regulate oocyte development and contributes to infertility issues owing to limited or excess nutrition.

## MATERIALS AND METHODS

### Reagents and resources

See Table S2 for detailed information on the reagents used in these studies and Table S3 for the specific genotypes used in each figure/panel. All raw data used in this study can be found in Table S4.

### Fly stocks

Flies used in actin quantification and IVEM experiments were maintained on cornmeal/agar/yeast food at room temperature except as noted below. Flies used in all other experiments were maintained on molasses/agar/yeast/malt extract/corn flour/soy flour food at room temperature except as noted below. Stocks used were *y^1^w^1^* [Bloomington *Drosophila* Stock Center (BDSC), #1495], Oregon R (BDSC, #5), *bmm^1^* (Grönke et al., 2003), *bmm-GFP* (BDSC, #94600, (Zhao et al., 2022), *pxt^f01000^* (Harvard Exelixis Collection; Thibault et al., 2004), *pxt^EY03052^* (BDSC, #15620), *mdy^QX25^* (BDSC, #5095), *Lsd-2^KG00149^* (BDSC, #13382), *sqh-EYFP-ER* (BDSC, #7195; LaJeunesse et al., 2004), *sqh-EYFP-Golgi* (BDSC, #7193; LaJeunesse et al., 2004).

### Immunofluorescence and fluorescent reagent staining

For Figs 1, 2, 7, 8, S4 and S5, the following method, referred to as Staining Method 1, was used. Adult female and male flies (to allow for mating) younger than 2 weeks old were fed dry yeast for 48 h at room temperature before ovary dissection in PBS-T (0.1% Triton X-100 in phosphate-buffered saline, PBS). Ovaries were fixed with 4.1% formaldehyde in PBS for 12 min at room temperature. Ovaries were washed with PBS-T, and follicles of desired stages were isolated using forceps and pin vises. Follicles were blocked in ovary block (10% bovine serum albumin, 0.1% Triton X-100, 0.02% sodium azide in PBS) overnight at 4°C. Primary antibodies were diluted in ovary block and incubated overnight at 4°C. The following primary antibodies were obtained from the Developmental Studies Hybridoma Bank (DSHB) developed under the auspices of the National Institute of Child Health and Human Development and maintained by the Department of Biology, University of Iowa (Iowa City, Iowa): mouse anti-Calnexin99A at 1:100 (Cnx99A 6-2-1, deposited by Munro, S.) and mouse anti-Golgin84 at 1:1000 (Golgin84 12-1, deposited by Munro, S.) (Riedel et al., 2016). Additionally, the following primary antibodies were used: rabbit anti-Pxt preabsorbed at 1:10 on *pxt^−/−^* ovaries in ovary block and used at 1:1000 (Spracklen et al., 2014). GFP was visualized directly. Samples were washed three times with PBS-T, and then protected from light for the remainder of the experiment. Samples were incubated in the following secondary antibodies diluted to 1:1000 in ovary block overnight at 4°C: goat anti-rabbit IgG Alexa Fluor 633 (Invitrogen), goat anti-mouse IgG Alexa Fluor 488 (Invitrogen), and goat anti-rabbit IgG Alexa Fluor 488 (Invitrogen). Samples were washed three times with PBS-T and then stained with the following reagents diluted in ovary block: Phalloidin Alexa Fluor 633 (Invitrogen) 1:150, 1 h at room temperature; Nile Red (1 mg/ml, Sigma-Aldrich) 1:50, 1 h at room temperature; LipidSpot 610 (1000×, Biotium) 1:100, 20 min at room temperature and Hoechst 33342 (1 mg/ml, Thermo Fisher Scientific) 1:1000, 20 min at room temperature. Samples were washed three times with PBS-T, and then mounted on coverslips in Aqua Polymount (PolySciences).

For Figs 3, 4 and S1 the following protocol, referred to as Staining Method 2, was used. Adult female and male flies (to allow for mating) within 24 h of eclosion were fed wet yeast paste for 72 h at room temperature before dissection in room-temperature Grace's medium (Lonza). Ovaries were fixed in 4% paraformaldehyde in Grace's medium for 10 min at room temperature. Ovaries were washed six times (10 min each) in antibody wash (0.1% bovine serum albumin in PBS-T). Samples were stained overnight at 4°C with Alexa Fluor 488, 568, or 647 Phalloidin (Invitrogen) at 1:250. Samples were then washed five or six times in PBS-T for 10 min each and stained with 4′6′-diamidino-2-phenylindole (DAPI, 5 mg/ml) at 1:5000 in PBS for 10 min. Samples were rinsed in PBS and mounted in 1 mg/ml phenylenediamine in 50% glycerol, pH 9 (Platt and Michael, 1983).

### Image acquisition and processing

Microscope images of fixed and stained *Drosophila* follicles were obtained using the following microscopes: Leica SPE confocal microscope with an ACS APO 20×/0.60 IMM CORR-/D or ACS APO 40×/1.5 oil CS objective (Leica Microsystems), Zeiss 700 confocal microscope or Zeiss 880 confocal microscope (Carl Zeiss Microscopy) using a Plan-Apochromat 20×/0.8 working distance (WD)=0.55 M27 objective, Zeiss 980 confocal microscope (Carl Zeiss Microscopy) using a Pln Apo 20×/0.8 or Pln Apo 40×/1.3 oil objective, or Leica SP5 confocal microscope (Leica Microsystems) using an HCX PL APO CS 63×/1.40 oil UV objective and Leica HyD detectors. Maximum projections of images, image cropping and image rotation were performed in Fiji/ImageJ software (Abramoff et al., 2004); scale bars were added in either Fiji, Adobe Illustrator or Adobe Photoshop and figures were assembled using Adobe Illustrator. Images in Fig. 4 and Fig. S1 were brightened by 30% in Adobe Photoshop to improve visualization.

### Quantification of actin defects

Confocal images of phalloidin-stained S10B follicles were collected as described. Actin bundle and cortical actin defects were scored by scanning through confocal *z*-stacks of S10B follicles in ImageJ by an operator unaware of the genotype. Representative images of follicles and scoring criteria are provided in Fig. S1. Briefly, actin bundles were scored as normal (score=0) if they were straight, forming from the nurse cell plasma membrane and oriented inwards toward the nurse cell nuclei. Mild defects in bundling (score=1) were defined as sparse bundle formation or slight delay in bundle growth relative to follicle development. Moderate defects (score=2) were defined as collapsed, thick and/or missing bundles from regions of the nurse cell plasma membrane. Severe defects (score=3) included those previously described, as well as a complete failure of bundles to form. Cortical actin was scored based on whether the cortical actin was intact or disrupted. Defects in cortical actin were evident by an absence or incomplete phalloidin staining between nurse cells and/or by nurse cell nuclei being in close proximity or contacting each other. Normal cortical actin (score=0) was defined as being completely intact and fully surrounding each nurse cell. The degree of severity of cortical actin defects was determined by the relative number of disruptions in cortical actin observed with mild defects (score=1) having a single instance, moderate defects (score=2) having two instances, and severe defects (score=3) having three or more instances of disrupted cortical actin. The ADI was then calculated by adding the bundle and cortical actin defect scores and binning them into normal (total score=0-1), mild defects (total score=2-3) or severe defects (score=4-6). Pearson's chi-squared analysis was performed using R (www.r-project.org).

### IVEM assays

For both the standard IVEM and modified IVEM assays, adult female and male flies (to allow for mating) within 24 h of eclosion were fed wet yeast paste daily for 3 days prior to dissection. Ovaries were dissected in room temperature IVEM or modified IVEM medium. IVEM medium: Grace's medium (Lonza), 1× penicillin/streptomycin (from 100× stock, Gibco) and 10% FBS (Atlanta Biologicals). Modified IVEM medium: Grace's medium, 1× penicillin/streptomycin and 2.5% FBS. S10B follicles were isolated and distributed between wells of a 24-well plastic tissue culture plate (Falcon) and 1 ml of fresh medium was added with or without additions (described below). Follicles were incubated overnight at room temperature in the dark. The next day, the number of follicles at each developmental stage were counted, and the percentage developing was calculated; follicles that reached S12 and older were considered developed. Statistical analysis was performed using the unpaired *t*-test function in Prism 8 or 9 (GraphPad Software).

For the exogenous PGF_2α_ analog [Fluprostanol (Flu), Cayman Chemical Company] experiments, the standard IVEM was performed and stock solutions of 10 µM Flu and 0.5 M aspirin (Cayman Chemical Company) were prepared in 100% ethanol. For each genotype, there were two wells of S10B follicles with one well treated with ethanol (control) and the other with Flu (final concentration of 20 nM). As an additional control, wild-type follicles were also treated with 1.5 mM aspirin to verify that Flu rescues the loss of PG synthesis; any experiment in which Flu failed to suppress the effects of aspirin was excluded from the analysis. In all conditions, the total amount of ethanol was kept constant. Follicle development was assessed as described above.

For the exogenous fatty acid (AA or OA) experiments, the modified IVEM was performed. AA and OA stock solutions were made in 100% ethanol, and total ethanol volumes were kept constant in each experiment. Follicle development was assessed as described above.

### Western blots

Follicles from well-fed females of the indicated genotypes and stage were dissected and fixed as described in Staining Method 1. Note that proteins from such samples ran at the same molecular weight as those from lysates prepared from live ovary tissue (Fig. S3). For each sample, 25-50 follicles were collected and boiled in sample buffer (Laemmli Sample Buffer with 2-mercaptoethanol; both from Bio-Rad). Samples were run on 4-15% gradient gels (Bio-Rad) at 80-120 V and transferred onto PVDF membrane (Immobilon-FL, EMD Millipore) in Towbin (10% Tris-Glycine+20% methanol; 80 V for 30 min; for anti-PLIN2, anti-Pxt, anti-Calnexin) transfer buffer. Membranes were blocked for 1 h at room temperature in LI-COR Odyssey Block (LI-COR Biosciences). Membranes were incubated overnight at 4°C with primary antibodies diluted in Odyssey Block. The following primary antibodies were used: rabbit anti-Pxt (1:5000; Spracklen et al., 2014), rabbit anti-PLIN2 (1:5000; Welte et al., 2005), mouse anti-Calnexin99A (1:500, DSHB) and mouse anti-α-Tubulin (1:5000, Cell Signaling Technology). Membranes were washed three times in PBS-0.1% Tween 20 (PBS-Tween). The following secondary antibody incubations were performed for 1 h at room temperature: IRDye 800CW goat anti-rabbit IgG (1:5000, LI-COR), and IRDy3 680RD goat anti-mouse IgG (1:5000, LI-COR). After secondary antibody incubation, membranes were washed twice in PBS-Tween and once in PBS. Membranes were imaged on a LI-COR Odyssey CLx imager and blots were processed and quantified in Image Studio Lite 5.2 (LI-COR). For quantifications, the fluorescence intensity of each band was measured and normalized to respective experimental controls. Data were analyzed and plotted using Prism (GraphPad).

### Lipid extractions

Twenty-five ovaries per sample were dissected from mated, mixed-age, and dry yeast-fed females and homogenized in a 2 ml Eppendorf tube with a motorized pestle (KONTES pellet pestle) in PBS and kept on ice. Lysates were then incubated 1:1 in a 2:1 chloroform/methanol mixture overnight at 4°C. Samples were spun in an Eppendorf Microcentrifuge (model 5415D) at maximum speed at room temperature for 1 min, and the bottom organic layer was transferred to a 0.65 ml Eppendorf tube. Samples were then vacuum-dried and either analyzed immediately or stored at −80°C for thin layer chromatography analysis, or before being shipped on dry ice for mass spectrometry analysis.

### Thin layer chromatography

Evaporated lipids were resuspended in 10 µl 2:1 chloroform/methanol and spotted on dehydrated silica plates (EMD Millipore; dehydrated by baking for 30 min at 100°C). Plates were placed in a chamber (Millipore Sigma/Sigma-Aldrich, Z266000) pre-saturated for 30 min with petroleum ether/diethyl ether/acetic acid (32:8:0.8) and allowed to develop until the solvent almost reached the top of the plates, and the solvent line was quickly marked with a pencil after removing plates from the chamber. The plates were then air-dried and briefly submerged in charring solution (50% ethanol, 3.2% H_2_SO_4_, 0.5% MgCl_2_). Plates were air-dried briefly and were charred for 30 min at 120°C. Plates were imaged using a Bio-Rad Gel Doc and bands were quantified using Fiji. Lipids were identified according to Knittelfelder and Kohlwein (2017) and by comparing whole lipid extracts to lipid standards (Millipore Sigma). Colorimetric TAG band intensities were quantified in Fiji. Bands were normalized to one condition on the plate (e.g. WT, Fig. 6F) to account for variability in charring from plate to plate. Paired Student's *t*-tests were run in GraphPad Prism for Fig. 6F.

### LC-MS/MS analysis

Extracted lipids were dissolved in corresponding volumes of 2:1 methanol:chloroform (v/v) and 5 μl of each sample was injected for positive and negative acquisition modes, respectively. Mobile phase A consisted of 3:2 (v/v) water/acetonitrile, including 10 mM ammonium formate and 0.1% formic acid, and mobile phase B consisted of 9:1 (v/v) 2- propanol/acetonitrile, also including 10 mM ammonium formate and 0.1% formic acid. Lipids were separated using an UltiMate 3000 UHPLC (Thermo Fisher Scientific) under a 90 min gradient; during 0-7 min, elution started with 40% B and increased to 55%; elution was then increased to 65% B from 7 to 8 min; maintained at 65% B from 8 to 12 min; increased to 70% B from 12 to 30 min; increased to 88% B from 30 to 31 min; increased to 95% B from 31 to 51 min; increased to 100% B from 51 to 53 min; maintained at 100% B during 53-73 min; then solvent B was decreased to 40% from 73 to 73.1 min, and then maintained for another 16.9 min for column re-equilibration. The flow-rate was set to 0.2 ml/min. The column oven temperature was set to 55°C, and the temperature of the autosampler tray was set to 4°C. Eluted lipids were analyzed using Orbitrap Q Exactive (Thermo Fisher Scientific) Orbitrap mass analyzer. The spray voltage was set to 4.2 kV, and the heated capillary and the HESI were held at 320°C and 300°C, respectively. The S-lens RF level was set to 50, and the sheath and auxiliary gas were set to 35 and 3 units, respectively. These conditions were held constant for both positive and negative ionization mode acquisitions. External mass calibration was performed using the standard calibration mixture every 7 days. MS spectra of lipids were acquired in full-scan/data-dependent MS2 mode. For the full-scan acquisition, the resolution was set to 70,000, the AGC target was 1e6, the maximum integration time was 50 ms, and the scan range was m/z=133.4-2000. For data-dependent MS2, the top ten ions in each full scan were isolated with a 1.0 Da window, fragmented at a stepped normalized collision energy of 15, 25 and 35 units, and analyzed at a resolution of 17,500 with an AGC target of 2e5 and a maximum integration time of 100 ms. The underfill ratio was set to 0. The selection of the top ten ions was subject to isotopic exclusion with a dynamic exclusion window of 5.0 s. MS data were analyzed using LipidSearch version 4.1 SP (Thermo Fisher Scientific). Identified lipid species with grade A and B were manually curated. A total of 98 different triglyceride species were identified (Table S1).

For the quantitative analysis in Fig. 6A-D,G,H and Fig. S5, four biological replicates containing 25 ovaries each from both wild-type and *ATGL* mutant females were analyzed and the quantity of the 98 triglyceride species determined, relative to background. One wild-type sample contained an order of magnitude less lipid and was discarded as an outlier (Table S1). In total, 25 different types of FAs were identified in the ovary triglycerides. The abundance of each FA was estimated by summing the amount of each triglyceride species containing that FA, weighted by how many times that FA is represented in that triglyceride. For Fig. 6A,B, this abundance is expressed as fraction of the total amount of fatty acids such identified. For Fig. 6C, the signal for all species of phosphatidylcholine, phosphatidylethanolamine and phosphatidylinositol, respectively, was summed and normalized to the average of the wild type. For Fig. 6D, the signal for all triglyceride species was summed and normalized to the average of the wild type. To express the abundance of various lipid species relative to total lipids recovered (Fig. S5), values for various lipid species were divided by the sum of all lipids in the sample. For Fig. 6G,H, the signal for two AA-containing triglyceride species was computed and normalized to the average of the wild-type signal. Amounts and fractions were calculated using Microsoft Excel and graphed using Prism 8 (GraphPad Software). Statistical tests for Fig. 6 were as follows: 6A and 6C was Šídák's multiple comparisons test, 6B was Tukey's multiple comparisons test, 6D was an unpaired, two-tailed *t*-test, and 6G,H were unpaired, two-tailed *t*-tests.

### Ovary centrifugation

Ovary centrifugations were performed as described (White and Welte, 2023), adapting a method previously described for embryos (Tran and Welte, 2010). Mated adult females of mixed ages fed dry yeast were anesthetized and beheaded before being mounted in microfuge tubes filled with 2.5% low melt agar. Tubes were spun for 10 min at 9000 ***g*** at 4°C. Flies were removed from agar with forceps and ovaries were isolated and fixed in 4% formaldehyde at room temperature for 15 min. Ovaries were washed three times in antibody wash for 5 min. Ovaries were stained with LipidSpot 610 (1000×, Biotium) 1:100 at room temperature for 20 min. Alternatively, ovaries incubated *in vitro* with fluorescently labeled AA were placed into the agar-filled microfuge tubes, covered with 10 µl maturation medium (see below), and centrifuged for 10 min at ∼6000 ***g*** at 4°C. Ovaries were recovered and treated as described in Staining Method 1. In the corresponding images, the orientation of centrifugation is indicated by an arrow that points towards the center of rotation.

### Lipid droplet purification

For each replicate, 1000-1200 ovaries were rapidly dissected from mated and dry yeast-fed females and kept on ice in TSS (68 mM NaCl, 0.03% Triton X-100) in 2 ml Eppendorf tubes. Ovaries were then washed three times with TKM (50 mM Tris, pH 7.4, 25 mM KCl, 5 mM MgCl_2_) to remove detergent. TKM was removed and the volume of ovaries was estimated visually. Ovaries were kept on ice, to which were added: two times the estimated ovary volume of TKM+1 M sucrose, protease inhibitor cocktail to final concentration of 1× protease inhibitor cocktail (Sigma-Aldrich), and calyculin A serine/threonine phosphatase inhibitor (10 µl per ml of volume; Cell Signaling Technology). Ovaries were homogenized on ice by grinding with an automated tissue grinder (KONTES pellet pestle) for 1-2 min, and then 20 µl ovary lysate samples were transferred from the total lysate to 0.65 ml Eppendorf tubes and stored at −80°C. The remaining ovary lysates were spun at ∼16,000 ***g*** for 10 min in an Eppendorf Microcentrifuge (model 5145D) at 4°C. The following was then added to samples slowly to avoid disturbing the LD layer: 300 µl TKM+0.5 M sucrose, 300 µl TKM+0.25 M sucrose, 400 µl TKM (no sucrose). Tubes were spun for 20 min at 4°C, with the speed adjusted as follows: ∼90 ***g*** for 5 min, ∼2200 ***g*** for 5 min, ∼16,000 ***g*** for 10 min. Purified LDs were then scooped off the top of the sucrose gradient using a drawn Pasteur pipette loop (Fisher Scientific, loop drawn by holding over a flame). LDs were washed off the pipette loop with 20 µL TKM (no sucrose) and stored at −80°C until analyzed.

In previous western analyses, we loaded equal amounts of protein from LD preparations and original tissue lysate for easy comparison (Li et al., 2012). This approach was not feasible here because of the relatively low amounts of LDs recovered from the large volume of ovaries used. Instead, we compared the LD sample to dilutions of ovary lysate. Purified LDs and ovary lysates were diluted as indicated in 2× Laemmli Sample Buffer before being boiled for 30 min and subsequently run on 4-15% SDS-PAGE gradient gels (western blot protocol as described above). Before the blocking step, the membrane was cut with a razor blade between 50 and 75 kDa and each half was treated separately. Quantification was performed as described above in western blots.

### Poly-D-lysine-coated dishes for live imaging

Poly-D-lysine (1 mg/ml, Gibco) was diluted 1:4 in PBS and 200 µl was added to each glass-bottom culture dish (MatTek Corporation). Dishes were incubated at 37°C for 1 h, the excess liquid was removed, and the dishes were allowed to dry.

### Fluorescent AA supplementation

A 2 mM chloroform/methanol stock solution of fluorescent AA {2-[(7-nitro-2-1,3-benzoxadiazol-4-yl)amino] AA (NBD AA), Avanti Polar Lipids} was kept at −80°C. Adult female and male flies (to allow for mating) younger than 2 weeks old were fed dry yeast for 48 h at room temperature or 24 h at 25°C in preparation for dissection. For *in vivo* supplementation, flies were fed supplemented yeast paste (1% sucrose, 5 µM NBD AA, dry yeast) overnight and follicles were dissected, fixed and stained as described in Staining Method 1. For *in vitro* supplementation, ovaries were dissected in maturation medium [Schneider's *Drosophila* medium, Sigma-Aldrich; 15% FBS, Gibco; 10 mg/ml insulin, Sigma-Aldrich; 1× penicillin/streptomycin (10,000 U/ml penicillin and 10 mg/ml streptomycin, pH 6.95), Gibco], and then S10B follicles were isolated and incubated for 15 min in maturation medium supplemented with 5 µM NBD AA and 2.5 µl of LipidSpot 610 (1000×, Biotium). The medium was removed by aspiration and the follicles were washed once in fresh maturation medium for 5 min. The follicles were then transferred to a poly-D-lysine-coated, glass-bottom culture dish with 200 µl of maturation medium. Follicles were allowed to adhere to the culture dish for 5 min prior to imaging.

## Supplementary Material

10.1242/develop.201516_sup1 Supplementary information
