# Supplementary material for: Adipose triglyceride lipase promotes prostaglandin-dependent actin remodeling by regulating substrate release from lipid droplets
Source: Development. 2023 Jun 8;150(20):dev201516. doi: 10.1242/dev.201516 (PMC10281261; doi:10.1242/dev.201516)
Supplement: Supplementary information [file develop-150-201516-s1.pdf]

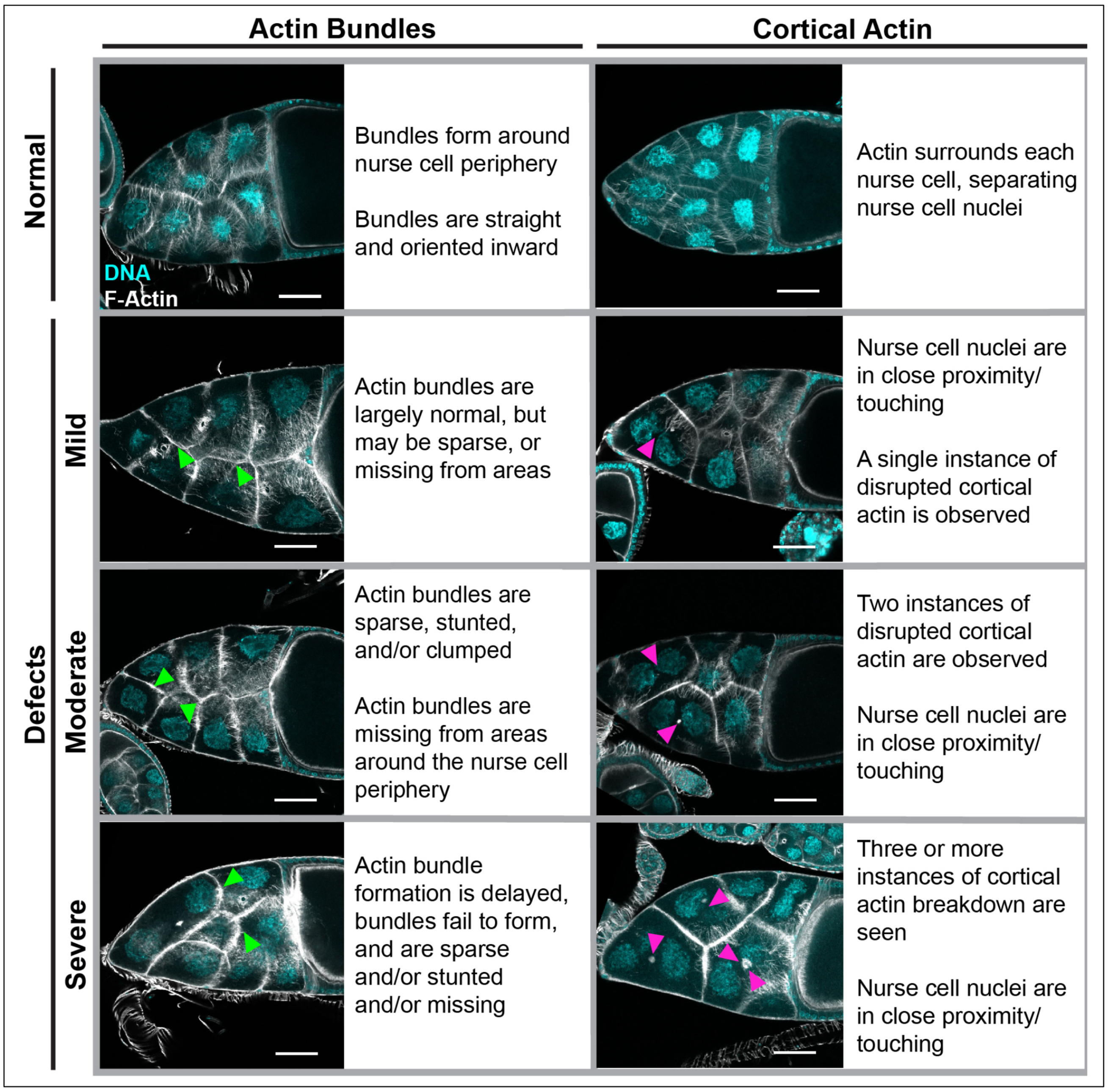

Fig. S1. Examples and criteria used for scoring actin remodeling phenotypes. Maximum projection of three confocal slices of representative S10B follicles stained for F-Actin (phalloidin) in white and DNA (DAPI) in cyan, and definitions of the indicated actin bundle and cortical actin phenotypes. Actin bundle and cortical actin phenotypes were independently binned into four categories: Normal, Mild Defects, Moderate Defects, or Severe Defects. Images were brightened by 30% to increase clarity. Arrowheads indicate examples of actin bundle defects (green), and cortical actin breakdown (magenta). Scale bars=50µm.

Development • Supplementary information

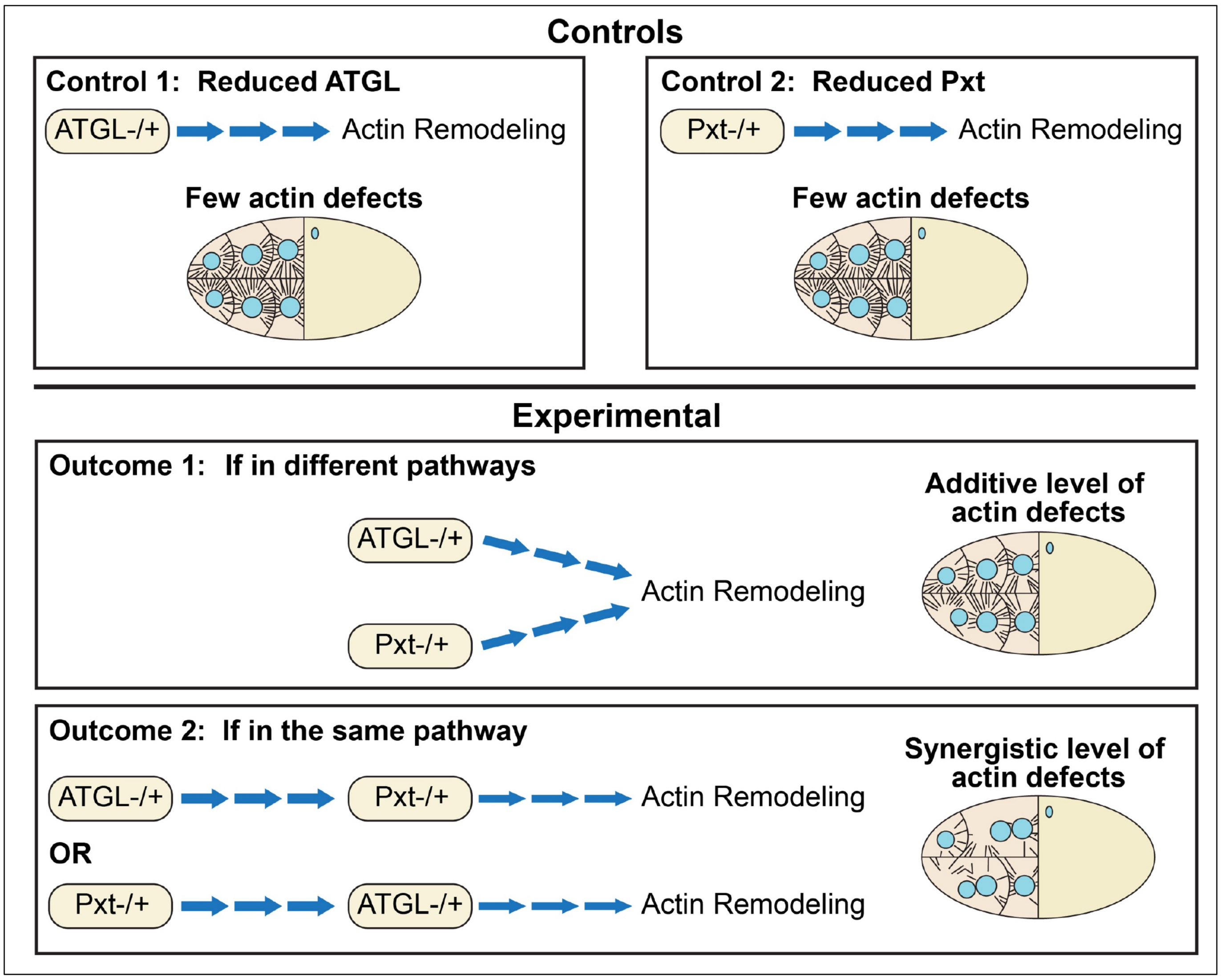

Fig. S2. Schematic of dominant genetic interactions.
Schematic of the premise of how dominant genetic interactions are used to determine if two factors act in the same pathway. Specifically shown are the controls and potential experimental outcomes for assessing if ATGL and Pxt function separately or together to control actin remodeling during S10B. The top depicts the Controls, single heterozygous follicles that have either reduced ATGL (**Control 1**) or reduced Pxt (**Control 2**); these control follicles are expected to have few actin defects. The bottom depicts the two possible experimental outcomes. **Outcome 1** depicts that ATGL and Pxt function in two separate pathways to regulate actin remodeling. In this case, the double heterozygous follicles will have actin defects that are similar to or additive of those seen in the control conditions. **Outcome 2** depicts that ATGL and Pxt function in the same pathway, with either ATGL acting upstream of Pxt or vice versa, to control actin remodeling. In this case, the double heterozygous follicles will exhibit a synergistic increase in actin defects that are more than additive of what is observed in the control conditions. The latter outcome is what is observed in Figure 4D-F.

Development • Supplementary information

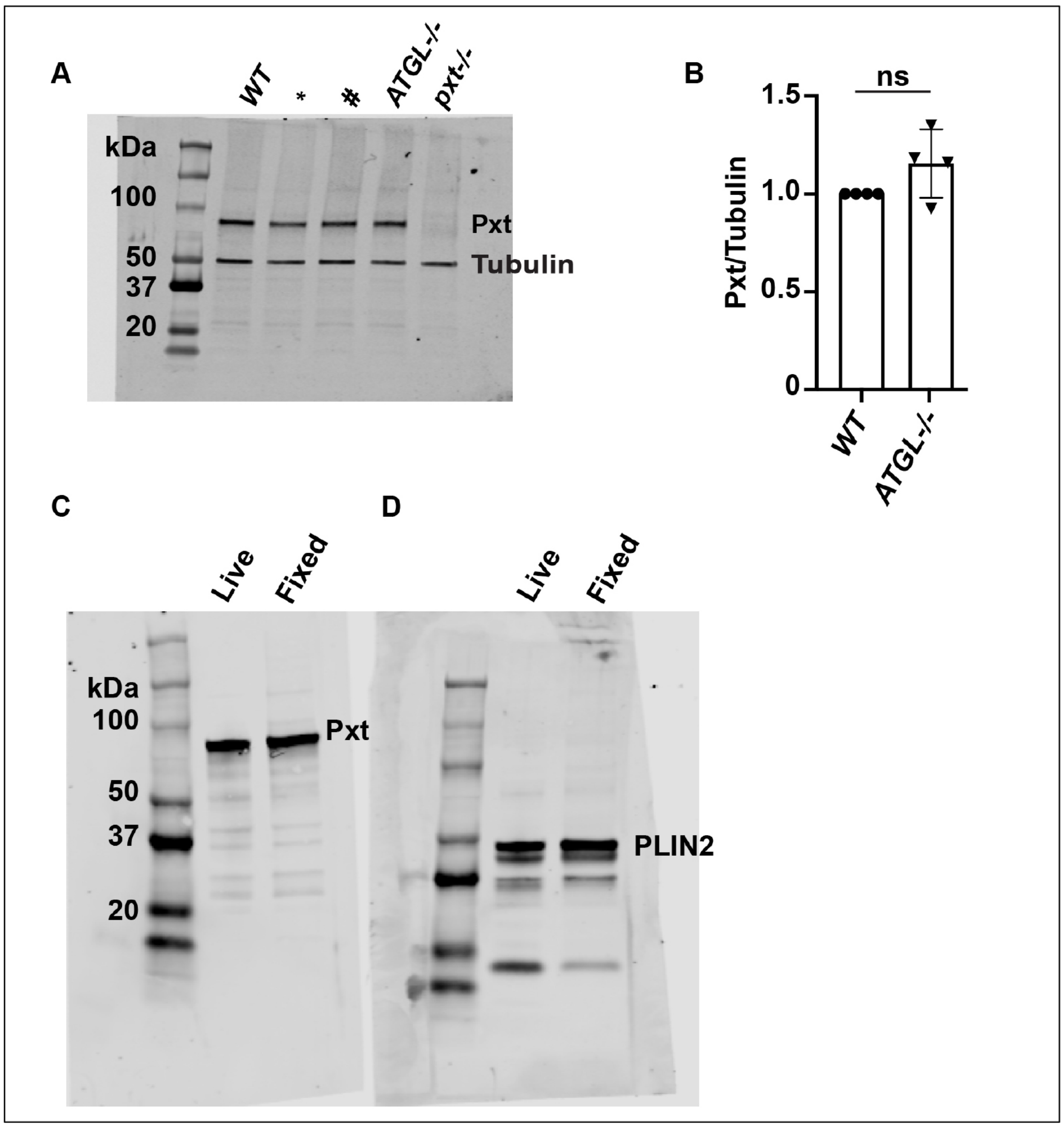

Fig. S3. ATGL does not affect Pxt expression.

(**A**) Immunoblot for Pxt and α-Tubulin of the indicated genotypes: WT, wild-type (Oregon R), *ATGL-/-* (*bmm*^{1}/*bmm*^{1}) and *pxt-/-* (*pxt*^{f01000}/*pxt*^{f01000}). The * and # lanes correspond to unrelated genotypes. (**B**) Quantification of **A**, in which band intensity was normalized as indicated. ns=p>0.05, unpaired t-test, two-tailed. (**C-D**) Immunoblots comparing the blotting patterns of live versus formaldehyde fixed wild-type whole ovary samples, 5 ovaries per lane, for Pxt (**C**) and PLIN2 (**D**). Loss of ATGL has no effect on Pxt levels (**A-B**). The live vs. fixed banding pattern is comparable for both Pxt (**C**) and PLIN2 (**D**) antibodies. Fixed samples were used in panel **A**.

Development • Supplementary information

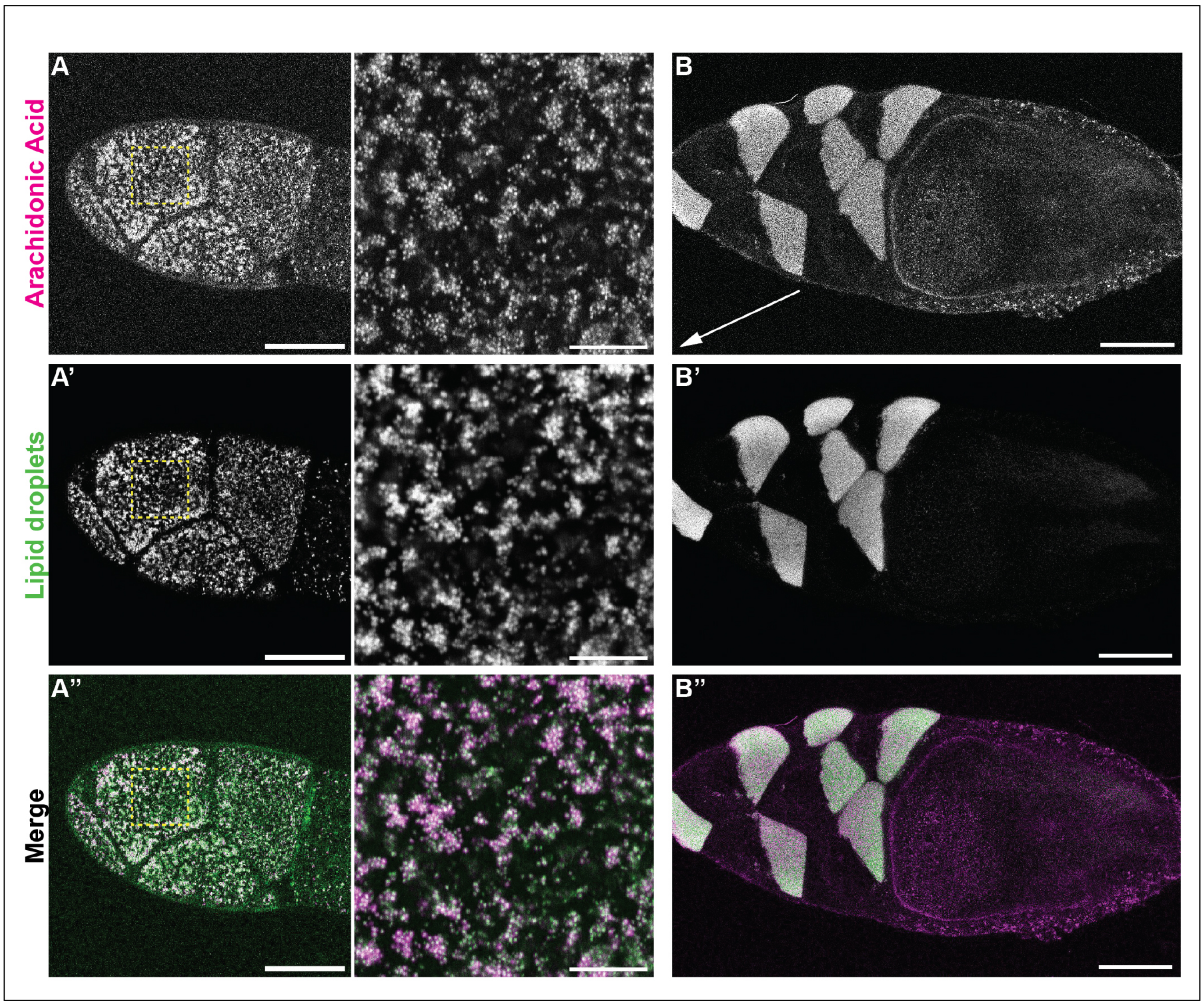

Fig. S4. AA from diet localizes to lipid droplets in Stage 10B follicles.
(**A-B''**) Confocal slice of live (**A-A''**, zoom-in of yellow boxed region) or (**B-B**) centrifuged wild-type (Oregon R) S10B follicles from flies fed 5µM NBD AA for ~12h, AA (green in merge) and LDs (LipidSpot, magenta in merge). In **A-A''** a focal plane near the nurse cell surface was chosen as LD density there is lower than in the center, making colocalization is easier to judge. In **B-B''** orientation of follicle during centrifugation is indicated by arrow. Scale bar = 50µm or 10 µm (zoomed in images in A-A''). AA provided in the diet localizes to LDs in S10B follicles (**A-B''**).

Development • Supplementary information

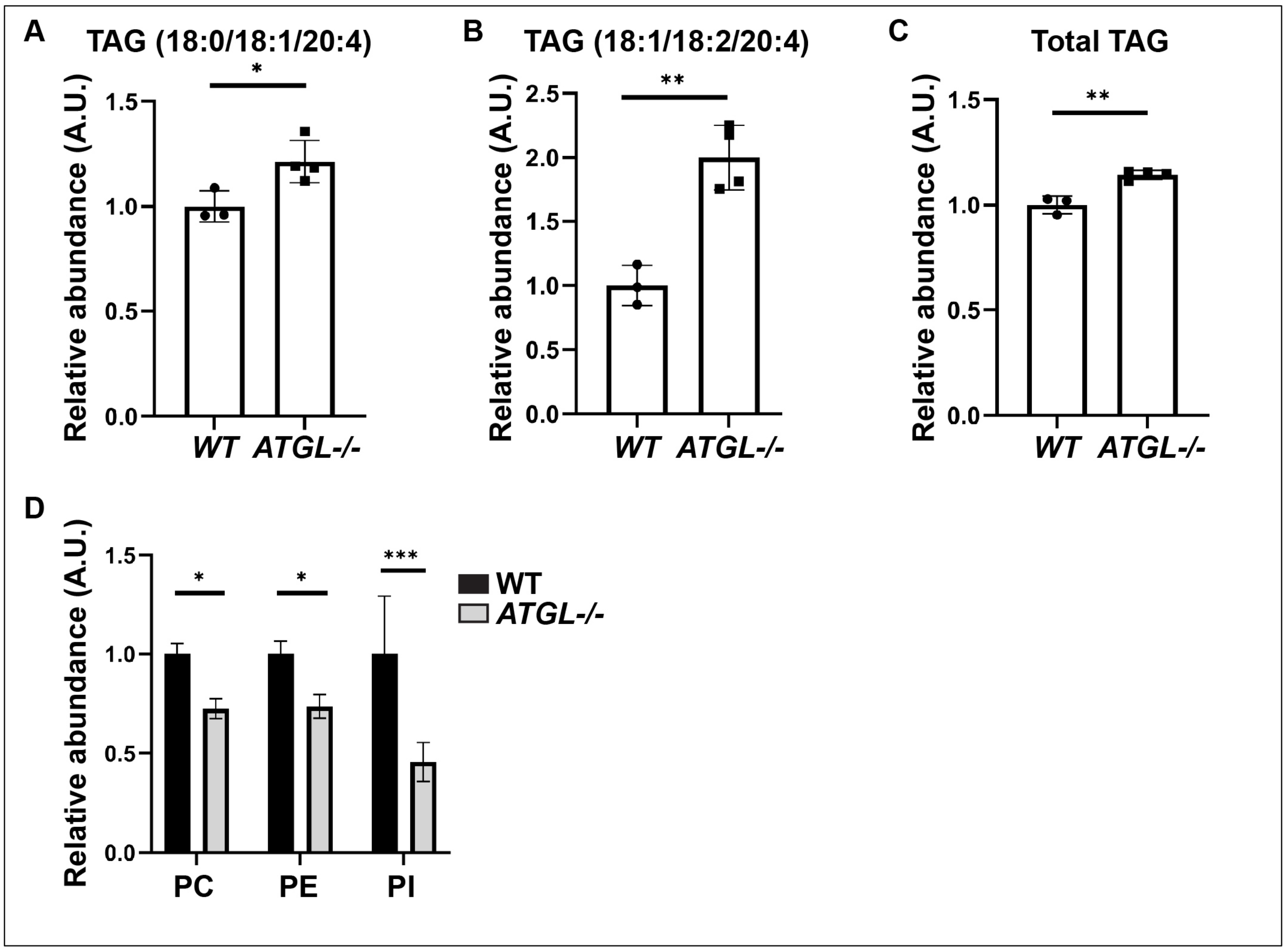

Fig. S5. AA-containing TAG and total TAG normalized to total lipids in sample.

(**A-D**) Lipids were extracted from wild-type (Oregon R) and *ATGL-/-* (*bmm[1]/bmm[1]*) ovaries and analyzed by mass spectrometry. Data from Figure 6 re-plotted as relative abundance normalized to total lipids in the sample. Error bars, SD. (**A, B**) Two triglyceride species containing arachidonic acid (AA). Error bars, SD, **p=0.0279, **p=0.0019*, unpaired t-tests, two-tailed. (**C**) Overall triglyceride levels are slightly increased in *ATGL-/-* ovary lipids. Error bars, SD, ***p=0.002*, unpaired t-test, two-tailed. (**D**) Relative amounts of phosphatidylcholine (PC), phosphatidylethanolamine (PE), and phosphatidylinositol (PI) in wild-type versus *ATGL-/-* ovary lipids. Error bars, SD, **p=0.0329, *p=0.0416, ***p=0.0001*, Sidak's multiple comparisons test. While overall triglyceride levels are similar between the two genotypes (**C**), the AA-containing triglycerides are increased (**A, B**), and three classes of phospholipids are decreased (**D**) in the absence of ATGL. The reason for the decrease in phospholipids is not clear. One possibility is that ATGL breaks down triglycerides to generate precursors for phospholipid production; alternatively, in the absence of ATGL, ovaries may contain a different mix of follicle stages (and thus different levels of triglyceride accumulation) due to altered developmental progression.

Development • Supplementary information

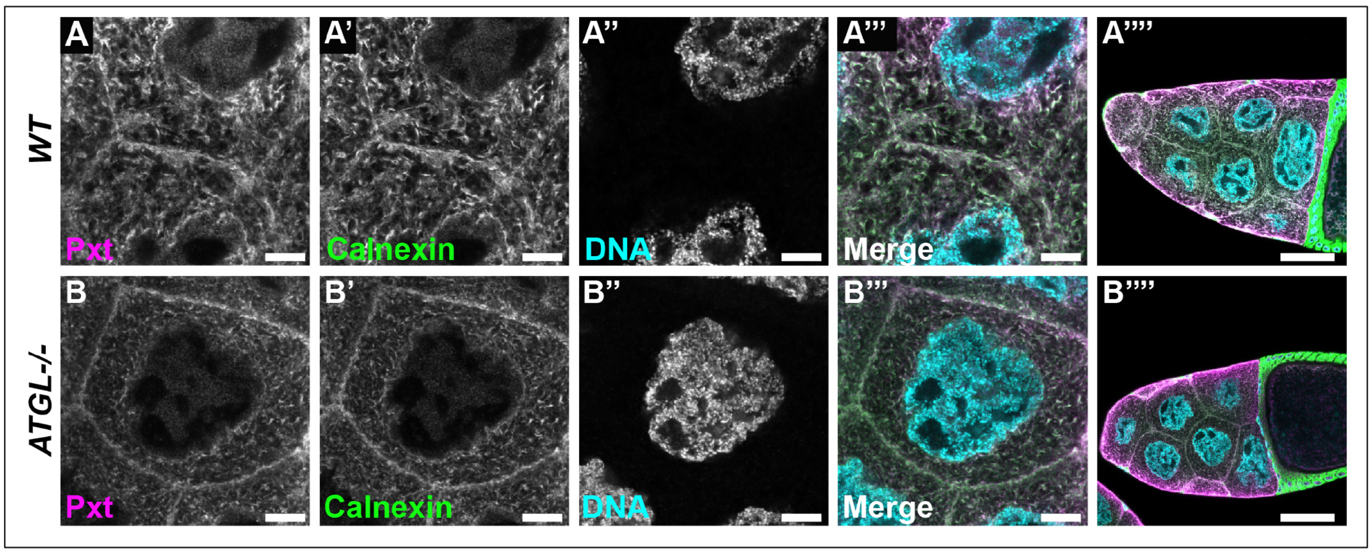

Fig. S6. ATGL does not affect Pxt's localization to the ER.

(**A-B''''**) Single confocal slice of S10B nurse cells of the indicated genotypes, stained for Pxt (**A-B**), Calnexin (**A', B'**, ER marker), and DNA (**A'', B''**, Hoechst). Merged images (**A'''-A'''', B'''-B''''**): Pxt, magenta; Calnexin, green; and DNA, cyan. Genotypes: WT, wild-type (Oregon R); *ATGL-/-* (*bmm^1/ bmm^1*), and *pxt-/-* (*pxt^{f01000}/pxt^{f01000}*). Scale bars = 10µm, except in A'''' and B'''' where = 50µm. Black boxes were added under panel and/or channel labels in **A, A'** and **A'''** to aid in visualization. In S10B follicles, Pxt's localization to the ER is similar to wild-type (**A-A'''**) in *ATGL* mutants (**B-B''''**).

Development • Supplementary information

Table S1. Quantitation of triglyceride species in ovaries

Column A lists the fatty acid content of various triglyceride species detected in ovaries by lipidomics. Columns B through H show the relative amount of those species in three wild-type (Oregon R) and four ATGL-/- (*bmm*^{1}/*bmm*^{1}) samples. Reads are background corrected. A fourth wild-type sample was discarded as an outlier because the detected lipid amounts were an order of magnitude lower than in the other samples.

Click here to download Table S1

Table S2. Key Resources Table.

Click here to download Table S2

Table S3. Genotype by figures.

List of genotypes shown in the figures. * indicates phenotypic example of genotype not related to the data presented in the manuscript.

Click here to download Table S3

Table S4. Raw data.

Raw data used for all quantifications presented in both the primary and supplemental figures.

Click here to download Table S4
