# Supplementary material for: Adipose triglyceride lipase promotes prostaglandin-dependent actin remodeling by regulating substrate release from lipid droplets
Source: Development. 2023 Jun 8;150(20):dev201516. doi: 10.1242/dev.201516 (PMC10281261; doi:10.1242/dev.201516)
Supplement: Supplementary file 1 [file develop-150-201516_review_history.pdf]

Adipose triglyceride lipase promotes prostaglandin-dependent actin remodeling by regulating substrate release from lipid droplets

Michelle S. Giedt, Jonathon M. Thomalla, Roger P. White, Matthew R. Johnson, Zon Weng Lai, Tina L. Tootle and Michael A. Welte

DOI: 10.1242/dev.201516

Editor: Thomas Lecuit

Review timeline

Original submission:	7 December 2022
Editorial decision:	21 February 2023
First revision received:	11 April 2023
Accepted:	3 May 2023

Original submission

First decision letter

MS ID#: DEVELOP/2022/201516

MS TITLE: Adipose triglyceride lipase promotes prostaglandin-dependent actin remodeling by regulating substrate release from lipid droplets

AUTHORS: Michelle S. Giedt, Jonathon M. Thomalla, Roger P. White, Matthew R. Johnson, Zon Weng Lai, Tina L. Tootle, and Michael A. Welte

I sincerely apologise for the delay before getting back to you. I have now received all the referees reports on the above manuscript, and have reached a decision. The referees' comments are appended below, or you can access them online: please go to BenchPress and click on the 'Manuscripts with Decisions' queue in the Author Area.

The overall evaluation is very positive and we would like to publish a revised manuscript in Development, provided that the referees' comments can be satisfactorily addressed. Please attend to all of the reviewers' comments in your revised manuscript and detail them in your point-by-point response. If you do not agree with any of their criticisms or suggestions explain clearly why this is so. If it would be helpful, you are welcome to contact us to discuss your revision in greater detail. Please send us a point-by-point response indicating your plans for addressing the referee's comments, and we will look over this and provide further guidance.

Reviewer 1

Advance summary and potential significance to field

This manuscript by Giedt et al. examines the potential links between triglycerides, arachidonic acid (AA), lipid droplets, and egg maturation and actin cortex function. The authors demonstrate that disruption of the enzyme that creates free AA leads to defects in actin formation which are highly similar to those observed after prostaglandin (PG) disruption. As PG requires AA for its synthesis, this suggests that AA may have roles outside of energetic consumption and function in cell signaling events. High levels of AA appear to be toxic and/or disrupt oogenesis, and they suggest that lipid droplets are a safe mechanism for the storage of AA in triglyceride form. The manuscript is fairly

© 2023. Published by The Company of Biologists under the terms of the Creative Commons Attribution License (https://creativecommons.org/licenses/by/4.0/).

simple, but it is well-written and analysis is appropriately performed. The imaging and genetic analysis is very convincing. The only perplexing aspect of the study is an explanation of why AA is processed in lipid droplets while it appears that PG synthesis likely occurs in the ER–the authors are clearly aware of this, but it would be good to hear a little more background and/or speculation on how this could occur. A few detailed comments are below:

Comments for the author

1) This is likely obvious to the authors, but would the processing of a triglyceride to create AA be enough to free AA from the lipid droplet? It seemed that (maybe) there was an assumption that this would create an increase of diffusible AA in the cytoplasm, but wasn't sure if this is the case and how diffusible AA is. Depending on AA's diffusible qualities, this seems like an easy explanation for how it could arrive at the ER but if there is less evidence for this having a bit more explanation of how it might traffic inside the cell would be useful (see point 3 as well)

2) A more minor comment – is it known how PG regulates actin function? Fascin and Enabled are mentioned, is this through regulation of their expression or at the level of protein function? No worries if this is not known, but it was an obvious question while reading the paper.

3) I believe the biogenesis and degradation of lipid droplets has been controversial, but what is the current best understanding of their lifecycle in the developing egg? It would be good to hear a little more about this – if they are generated at the ER, could these incipient LDs be the relevant population given the conclusion of where the PG-creating enzyme is located? Other speculation on transient interactions with the ER, etc would be good to hear.

4) Line 299-301 seems that it should be softened a little – it is always hard to know if the strongest sites of localization obscure low level (or transient) recruitment to an alternative site of activity (primary populations may not be coincidental but small fractions could be – see how Myosin II levels in lamellipodia were missed for years because of how low level they are compared to other locations in the cell).

5) I think this probably would have been mentioned, but can ATGL act on any other lipid species? If so, are these species interconvertible with AA? Would be good to have a statement on this in the manuscript.

6) It is a well-put together manuscript – kudos to the authors.

Reviewer 2

Advance summary and potential significance to field

In this manuscript, the authors reveal a novel function for lipid droplets (LD) in Drosophila oogenesis by dissecting the molecular pathway upstream of the production of prostaglandin (PG). The Tootle lab previously demonstrated that PG is necessary to trigger the assembly of the nurse cell actin cable arrays and cortical actin that are required for the completion of oogenesis. While it was known LDs develop late in oogenesis, are transported into the oocyte during dumping, and are required to support embryogenesis, it was not known whether/how they also play a role in oogenesis itself. This work clearly demonstrates a critical role for LDs in oogenesis. Overall, the work is novel and significant and the manuscript presents compelling evidence for their final model. My comments are minor and likely can be addressed by changes to text and figures alone.

Comments for the author

Fig 1K,L lines 120-123 What is the basis for saying that there is a large predominance of triglycerides? What is the significance of plotting the TAG/Cholesterol ratio? I think more explanation of these data is needed.

Fig 2A I think the insets would be more useful at a significantly higher zoom.

© 2023. Published by The Company of Biologists under the terms of the Creative Commons Attribution License (https://creativecommons.org/licenses/by/4.0/).

Fig 3A-C Please include high mag images of the defects you are describing. There's more detail in Fig S1, but it's still difficult to visualize what is being described.

Fig 3F Add "ADI" to the figure to make it more consistent with the text.

Lines 169-174 It seems more intuitive to talk directly about the frequency of defects, rather than the frequency of normal follicles and then interpreting in the context of defects. Looking at the data in Fig 4, it seems clear that the cortical actin defects in the double mutants aren't merely additive, but that's less clear for the actin bundle defects. I think that some brief discussion of the nuances in these data is a good idea to have in this part of the results section.

Line 180 If there's precedent for actin regulators being sequestered in LDs please indicate.

Fig S3 panel D is not described completely in the legend.

Fig S4 lines 225-226 Please include higher mag images that make the co-staining clearly visible.

Fig 6 Lines 209-241 I found this section difficult to follow. I suggest presenting the fluorescent AA experiment first then describing the experiments that directly address AA levels and AA-containing TAGs in control and mutants.
Perhaps then finish the section with the data about bulk turnover not being affected, pointing to the potential specificity for lipid signaling. If the amount of TAG-AA is elevated in ATGL mutants, then I would predict that there would be less free AA in the mutant but that's not what you see (Fig 6A). Please explain.

Lines 243-282 Is it an undisputed fact that PGs are derived from only AA? I was surprised that the authors didn't show that adding AA to the medium could suppress the loss of ATGL in their IVEM assay. It seems like this result would significantly strengthen the argument that regulating AA level specifically is critical to the downstream signaling events. The authors did a version of this experiment (Fig 7D,F), but it was designed to address a different question about the relationship between ATGL function and AA level. If the experiment isn't needed, please justify.

I'm also a bit confused about the idea that free AA is "rapidly sequestered into LDs". If ATGL is localized to the LDs, then it seems that the LD is the site of AA production normally. Please clarify in light of the experiments in Fig. 7.

Fig 8 A-C Please provide high mag insets to more clearly demonstrate the localization of Pxt relative to the labeled structures.

Fig 8 D,E Please indicate the stage—presumably 10B based on the immunofluorescence? Also, please describe the data more completely.

Fig 9 Although already known, I suggest adding PG and the relevant actin-associated factors to the model for the sake of clarity and completion.

Discussion
Lines 307-308 If follicles die by S8/9 in DGAT1 mutants, didn't that already strongly suggest that LDs play a role in oogenesis?

Line 329 Why "conversely"?

© 2023. Published by The Company of Biologists under the terms of the Creative Commons Attribution License (https://creativecommons.org/licenses/by/4.0/).

First revision

Author response to reviewers' comments

We thank the reviewers for their enthusiasm for the study and for their suggestions for improving the manuscript. We have revised the manuscript accordingly. Major changes to the manuscript are shown in blue text in the file named: Manuscript_blue. We also provide a clean version of the manuscript, Manuscript_clean. Below, we detail the revisions and provide additional clarifications (in blue) to each of the reviewer comments (in black).

Reviewer 1:
1. This is likely obvious to the authors, but would the processing of a triglyceride to create AA be enough to free AA from the lipid droplet? It seemed that (maybe) there was an assumption that this would create an increase of diffusible AA in the cytoplasm, but wasn't sure if this is the case and how diffusible AA is. Depending on AA's diffusible qualities, this seems like an easy explanation for how it could arrive at the ER, but if there is less evidence for this having a bit more explanation of how it might traffic inside the cell would be useful (see point 3 as well)

PGs are produced at picogram quantities, therefore very little release of AA from triglycerides is needed for PG synthesis. It is unclear how AA moves from compartment to compartment in the cell. Instead, we predict AA is released by ATGL from LDs in close proximity to ER containing Pxt. We have added the following to the discussion:

Lines 353-363:
LDs originate from the ER and, in many cells, retain physical connections with the ER after biogenesis (Walther and Farese, 2012; Olzmann and Carvalho, 2019)}; in particular, the phospholipid monolayer around LDs can remain continuous with one leaflet of the ER membrane (Cottier and Schneiter, 2022). In addition, lipidic bridges between ER and LDs can form transiently. These connections can mediate exchange of proteins and lipids (Salo *et al.*, 2019; Cottier and Schneiter, 2022; Olarte *et al.*, 2022) and thus presumably transfer AA freed from LDs. What remains unclear is how far free AA can diffuse (either via the cytosol or through membranes) before being metabolized; it might only be able to reach Pxt molecules present in ER regions immediately adjacent to LDs. Nevertheless, LD-derived AA could still reach distant regions of the ER by being incorporated into ER phospholipids, which can diffuse throughout the entire ER; in a second step, AA would then be released by cPLA2 to provide substrate for Pxt.

2. A more minor comment – is it known how PG regulates actin function? Fascin and Enabled are mentioned, is this through regulation of their expression or at the level of protein function? No worries if this is not known, but it was an obvious question while reading the paper.

We have previously shown that PGs regulate the activity of actin binding proteins, not their expression. We have added this information to the introduction (Lines 90-93).

3. I believe the biogenesis and degradation of lipid droplets has been controversial, but what is the current best understanding of their lifecycle in the developing egg? It would be good to hear a little more about this – if they are generated at the ER, could these incipient LDs be the relevant population given the conclusion of where the PG-creating enzyme is located? Other speculation on transient interactions with the ER, etc would be good to hear.

There is lots of ongoing work on the biogenesis and turnover of LDs. Given the length constraints for the manuscript, we opted to not do an extensive literature review, but simply summarize the most pertinent information in the discussion under "Site of PG synthesis". In many cells, LDs remain connected to the ER via lipidic bridges that allow exchange of proteins and lipids. The idea by the reviewer that it might be in particularly the incipient LDs that drive PG synthesis is a good one; however, since even mature LDs are thought to remain connected to the ER, we did not elaborate on this in the text. Also see our response to point 1.
4. Line 299-301 seems that it should be softened a little – it is always hard to know if the strongest sites of localization obscure low level (or transient) recruitment to an alternative site of activity (primary populations may not be coincidental but small fractions could be – see how Myosin II levels

© 2023. Published by The Company of Biologists under the terms of the Creative Commons Attribution License (https://creativecommons.org/licenses/by/4.0/).

in lamellipodia were missed for years because of how low level they are compared to other locations in the cell).

We did not mean to imply there is no Pxt localizing to LDs. We have modified the language and state: “We conclude that the majority of Pxt is not on LDs. Thus, strong enrichment of the PG synthesis machinery on LDs is not required for LDs to play a role in PG production.” (Lines 303-305)

5. I think this probably would have been mentioned, but can ATGL act on any other lipid species? If so, are these species interconvertible with AA? Would be good to have a statement on this in the manuscript.

ATGL is highly specific for cleaving fatty acids from triglycerides. We have ensured this is clearly stated throughout the manuscript.

6. It is a well-put together manuscript – kudos to the authors.

We greatly appreciate the reviewer’s enthusiasm for the manuscript.

Reviewer 2:

1. Fig 1K,L lines 120-123 What is the basis for saying that there is a large predominance of triglycerides? What is the significance of plotting the TAG/Cholesterol ratio? I think more explanation of these data is needed.

We thank the reviewer for this insightful question. We had used cholesterol as an internal control but realize now that this is based on flawed reasoning since we have no evidence that cholesterol levels are constant across stages. We are now showing the raw TAG measurements instead and have deleted the claim about predominance of triglycerides because it is not clear how linear the detection in Fig. 1K is for different lipid species.

The corrected text now states (lines 116-118): “Using thin-layer chromatography, we find that developing follicles accumulate both classes of neutral lipids and triglyceride accumulation measured biochemically mirrors LD accumulation detected by microscopy (Fig. 1K-L).”

2. Fig 2A I think the insets would be more useful at a significantly higher zoom.

We added zoomed-in images as requested.

3. Fig 3A-C Please include high mag images of the defects you are describing. There’s more detail in Fig S1, but it’s still difficult to visualize what is being described.

We added zoomed-in images as requested.

4. Fig 3F Add “ADI” to the figure to make it more consistent with the text.

We added ADI to the y axis in both Fig. 3F and Fig. 4F.

5. Lines 169-174 It seems more intuitive to talk directly about the frequency of defects, rather than the frequency of normal follicles and then interpreting in the context of defects. Looking at the data in Fig 4, it seems clear that the cortical actin defects in the double mutants aren’t merely additive, but that’s less clear for the actin bundle defects. I think that some brief discussion of the nuances in these data is a good idea to have in this part of the results section.

Thank you for pointing out that we neglected to discuss these important differences. We have revised the text to state (Lines 162-166): Strikingly, the double heterozygotes have severe actin defects (Fig. 4A-F). Single heterozygotes (ATGL-/+ or pxt-/+) exhibit mild defects in actin bundles, with ~7% being severe, whereas double heterozygotes (pxt/ATGL) exhibit ~22% with severe bundle defects (Fig. 4D). Cortical actin defects are rare in the single heterozygotes, with over 87% being normal, conversely only 52% of the double heterozygotes are normal (Fig. 4E).

© 2023. Published by The Company of Biologists under the terms of the Creative Commons Attribution License (https://creativecommons.org/licenses/by/4.0/).

6. Line 180 If there's precedent for actin regulators being sequestered in LDs, please indicate.

We have revised the manuscript to state (Lines 175-178): "For example, PGs produced by Pxt might control ATGL levels or promote binding of actin regulators to ATGL and thus to LDs; indeed, actin binding proteins localize to LDs in various cell types (Pfisterer et al., 2017; Bersuker et al., 2018; Tan et al., 2021)."

7. Fig S3 panel D is not described completely in the legend.

Thank you for drawing our attention to this. We have revised the legend to state: "The live vs. fixed banding pattern is comparable for both Pxt (**C**) and PLIN2 (**D**) antibodies."

8. Fig S4 lines 225-226 Please include higher mag images that make the co-staining clearly visible.

We added zoomed-in images as requested.

9. Fig 6 Lines 209-241 I found this section difficult to follow. I suggest presenting the fluorescent AA experiment first then describing the experiments that directly address AA levels and AA-containing TAGs in control and mutants. Perhaps then finish the section with the data about bulk turnover not being affected, pointing to the potential specificity for lipid signaling. If the amount of TAG-AA is elevated in ATGL mutants, then I would predict that there would be less free AA in the mutant but that's not what you see (Fig 6A). Maybe we need to be clearer that these are the fatty acids from TAG, not free fatty acids. Please explain.

10. Lines 243-282 Is it an undisputed fact that PGs are derived from only AA? I was surprised that the authors didn't show that adding AA to the medium could suppress the loss of ATGL in their IVEM assay. Well….that's how the experiment started off. It seems like this result would significantly strengthen the argument that regulating AA level specifically is critical to the downstream signaling events. The authors did a version of this experiment (Fig 7D,F), but it was designed to address a different question about the relationship between ATGL function and AA level. But this is where it ended up. If the experiment isn't needed, please justify.

11. I'm also a bit confused about the idea that free AA is "rapidly sequestered into LDs". If ATGL is localized to the LDs, then it seems that the LD is the site of AA production normally. Please clarify in light of the experiments in Fig. 7.

As points 9-11 are highly inner-related, we addressed them together by significantly revising both "AA is present in ovary triglycerides" and "ATGL promotes AA release from LDs" to clarify the rationale (Lines 205-287).

In short, AA rescue is not feasible because it is rapidly sequestered into LDs, and excess free AA by supplying it exogenously is toxic. AA can also be stored in phospholipids. AA is the primary substrate for PG production, and AA release from cellular lipids (i.e. triglycerides or phospholipids) is tightly regulated and is the rate limiting step in PG production. The majority of prior studies on PGs indicate that phospholipids are the primary source of AA for PG production.

12. Fig 8 A-C Please provide high mag insets to more clearly demonstrate the localization of Pxt relative to the labeled structures.

We added zoomed-in images as requested.

13. Fig 8 D,E Please indicate the stage—presumably 10B based on the immunofluorescence? Also, please describe the data more completely.

The experiments used ~1000-1200 whole ovaries. This information has been added to the Figure legend.

© 2023. Published by The Company of Biologists under the terms of the Creative Commons Attribution License (https://creativecommons.org/licenses/by/4.0/).

14. Fig 9 Although already known, I suggest adding PG and the relevant actin-associated factors to the model for the sake of clarity and completion.

To avoid overcomplicating the model, we added PG and ABPs (explained in the legend) to the figure.

15. Discussion Lines 307-308 If follicles die by S8/9 in DGAT1 mutants, didn't that already strongly suggest that LDs play a role in oogenesis?

Death at S8/9 is a known nutritional/resource checkpoint in *Drosophila* oogenesis, which ensures that eggs are not produced without sufficient resources for embryonic viability. Thus, the arrest of *dgat1* mutant follicles could be due to the necessity of LDs for embryogenesis, and have nothing to do with a role of LDs in follicle development.

We state (Lines 313-314): "Our data reveal that the LDs produced during Drosophila oogenesis are not just lipid and protein stores for the future embryo but play a critical regulatory role during follicle development."

16. Line 329 Why "conversely"?

We have removed the word.

Second decision letter

MS ID#: DEVELOP/2022/201516

MS TITLE: Adipose triglyceride lipase promotes prostaglandin-dependent actin remodeling by regulating substrate release from lipid droplets

AUTHORS: Michelle S Giedt, Jonathon M Thomalla, Roger P White, Matthew R Johnson, Zon Weng Lai, Tina L Tootle, and Michael A Welte
ARTICLE TYPE: Research Article

I am happy to tell you that your manuscript has been accepted for publication in Development, pending our standard ethics checks.

Reviewer 2

Advance summary and potential significance to field

From the original review: "In this manuscript, the authors reveal a novel function for lipid droplets (LD) in Drosophila oogenesis by dissecting the molecular pathway upstream of the production of prostaglandin (PG). The Tootle lab previously demonstrated that PG is necessary to trigger the assembly of the nurse cell actin cable arrays and cortical actin that are required for the completion of oogenesis. While it was known LDs develop late in oogenesis, are transported into the oocyte during dumping, and are required to support embryogenesis, it was not known whether/how they also play a role in oogenesis itself. This work clearly demonstrates a critical role for LDs in oogenesis. Overall, the work is novel and significant, and the manuscript presents compelling evidence for their final model."

Comments for the author

The authors have clearly and thoroughly addressed all of my comments and questions. I highly support the publication of this manuscript in Development.

© 2023. Published by The Company of Biologists under the terms of the Creative Commons Attribution License (https://creativecommons.org/licenses/by/4.0/).
